# Network Pharmacology Combined with Animal Models to Investigate the Mechanism of ChangPu YuJin Tang in the Treatment of Tourette Syndrome

**DOI:** 10.2174/0113862073295447240430113053

**Published:** 2024-05-03

**Authors:** Man-Qi Lu, Zheng-Gang Shi, Jing Shang, Lei Gao, Wei-Jiao Gao, Lü Gao

**Affiliations:** 1 Gansu University Of Chinese Medicine Clinical College of Chinese Medicine, Lanzhou, 730000, China;; 2 Shanxi University Of Chinese Medicine Third Clinical Medical College Pediatric Teaching and Research Department, Taiyuan 140100, China

**Keywords:** Tourette syndrome, compound prescription of Chinese medicine, Chang Pu Yu Jin Tang, network pharmacology, BDNF signaling pathway

## Abstract

**Background:**

ChangPu YuJin Tang (CPYJT) is a Chinese herbal formula that has been shown to be an effective therapeutic strategy for pediatric patients with Tourette Syndrome (TS). Using an integrated strategy of network pharmacology and animal model, the aim of this study was to investigate the mechanism of CPYJT in the treatment of TS.

**Methods:**

Compound libraries of CPYJT were established using databases, such as the Traditional Chinese Medicine Systems Pharmacology Database and Analysis Platform (TCMSP). The TCMSP database and Swiss Target Prediction database were used to predict the targets. The above results were constructed into a CPYJT-Drug-Component-Target network. Moreover, TS targets were predicted using GeneCards and other databases. The targets corresponding to the potential ingredients in CPYJT and the targets corresponding to TS were taken as the intersections to construct the CPYJT-TS network. The target network was analysed by PPI using the string database. GO and KEGG enrichment analyses were performed on the target network. The whole process was performed using Cytoscape 3.7.2 to make visual network diagrams of the results. CPYJT was characterised by Ultra-Performance Liquid Chromatography-Tandem Mass Spectrometry (UHPLC-MS). Transmission Electron Microscopy (TEM) was used to observe the structural changes of CPYJT on the neuronal cells of the IDPN model rats. RT-PCR and Western Blot were used to analyse the changes in the mRNA and protein expression levels of BDNF, TrkB, PI3K, and AKT in the cortex, striatum, and thalamus brain regions after CPYJT administration in IDPN model rats.

**Results:**

Network pharmacology and UHPLC-MS studies revealed that CPYJT acted on the TS through multiple neurotransmitters and the BDNF/TrkB and PI3K/AKT signalling pathways. CPYJT ameliorated neurocellular structural damage in the cortex, striatum, and thalamus of TS model rats. Additionally, CPYJT up-regulated the levels of BDNF, TrkB, PI3k, and AKT in the cortex, striatum, and thalamus of TS model rats.

**Conclusion:**

It was found that CPYJT protected neuronal cells from structural damage in multiple brain regions and affected the expression levels of BDNF, TrkB, PI3K, and Akt in the cortex, striatum, and thalamus during TS treatment.

## 1 INTRODUCTION

Tourette Syndrome (TS), also known as Gilles de la Tourette’s Syndrome (GTS) or Tourette Disorder (TD), is a common neuropsychiatric disorder during childhood. Clinical manifestations are progressive development and various forms of exercise or (and) vocal tics [1]. TD occurs universally across all races, ethnicities, and populations. Transient and mild tic, chronic TD, and TS affect 20%, 0.3-5.0%, and 0.3-1.0% of school-age children in different Western populations, respectively [2, 3]. A meta-analysis in China reported that the incidences of transient TD, chronic TD, and TS from 1992 to 2010 were 1.7%, 1.2%, and 0.3%, respectively [4]. Tic symptoms or comorbidity seriously affect the quality of life and healthy growth of children [5]. Longitudinal studies have reported that about one-third of children with TS have symptoms that persist into adulthood [6].

TS mechanisms are unknown, and current research can be divided into two broad categories: structural nervous system abnormalities and functional alterations. Children with Tourette syndrome have intricate and widespread developmental problems throughout various parts of the brain. Structural abnormalities are common in the cortico-striato-pallido-thalamic white matter pathways [7]. Tic severity is linked to cortical thinning [8]. Neurophysiological studies have identified disruptions in functional connectivity in TS patients [9], but it is uncertain whether these are linked to neuronal cell damage or structural damage to myelinated mitochondria. CPYJT has been used in clinical practice for over 20 years, with apparent clinical efficacy in TS [10]. The current study aims to investigate whether CPYJT can mitigate ultrastructural damage to nerve cells, facilitate the restoration of nerve cell numbers, and provide therapeutic benefits in treating the disease, building on prior research demonstrating CPYJT's capacity to safeguard nerve cells and impede the decline in nerve cell count in TS model rats [11, 12].

It was found that hypermetabolism of DA and defective metabolism of the 5-HT system were significantly abnormal in TS but could not explain the onset of the disease in all children. Traditional Chinese medicine is effective in treating TS [13]. The effectiveness of the Chinese medicinal formula Paeonia lactiflora is comparable to the primary medication recommended in the Tourette's syndrome guideline, Tiapride tablets, but with a superior safety profile. This formula has been included in the European Clinical Guidelines for Tourette Syndrome and other related disorders [14], as published by the European Society for the Study of Tourette's Syndrome (ESSTS) in 2021. Network pharmacology is a branch of pharmacology that employs a network-based method to study the combined effects of medications, diseases, and targets. Traditional Chinese Medicine (TCM) is a complex system that utilizes several components, targets, and pathways to treat ailments with single medications or formulae, emphasizing a holistic approach. Network pharmacology is efficient in identifying bioactive compounds and explaining the mechanism of action of Traditional Chinese Medicine formulations [15]. Several experimental research works on CPYJT have also been conducted in the early stages, and it has been found to have a modulating effect on neurotransmitters, such as dopamine (DA), 5-hydroxytryptamine (5-HT), norepinephrine (NE) [16, 17]. Network pharmacological and compositional analyses can clarify whether CPYJT is able to act on TS through other targets and pathways. Based on the characteristics of the multi-targets of traditional Chinese medicine prescriptions, the present study will experimentally validate the theoretical pathways of action based on the network pharmacology study [18, 19].

## 2 MATERIALS AND METHODS

### 2.1 Network Pharmacology Studies

#### 2.1.1 CPYJT-Drug-Component-Target Network Construction

The TCMSP, SymMap, ETCM, and CACS databases were searched to collect 12 flavors of drug components to obtain the CPYJT-Drug-Component Network [20]. The targets of the ingredients were predicted using the target prediction system in TCMSP, and the above target results were evaluated and screened based on the criteria of oral bioavailability (OB) >30% and drug-like properties (DL) >0.18 [21-23]. A number of TCMs not listed in the TCMSP database were retrieved using the SwissADME function of the Swiss Target Prediction Database, which requires that both OB and DL conditions be met [24, 25]. The network of CPYJT, 12_Flavored drugs, ingredients, and targets was constructed. Cytoscape 3.7.2 was used to make a visual network diagram of the results.

The disease name “Tourette syndrome” was searched in GeneCards, DrugBank, and OMIM databases to collect disease therapeutic targets [26, 27].

#### 2.1.2 Network Construction of CPYJT-TS

The targets corresponding to the potential ingredients in CPYJT and the targets corresponding to TS were taken as the intersections. The CPYJT-TS network could help identify CPYJT effective protein targets and understand the mechanism of action of multicomponents and multitargets in TCM. The network was used for the enriched analysis of PPI, GO, and KEGG [28].

#### 2.1.3 CPYJT-TS Network Protein-Protein Interaction (PPI) Network Analysis

The gene symbols of the targets were used to assess the direct physical and functional interactions of the proteins by generating PPI network maps online using the STRING (version 11.5) database (https://version-11-5.string-db.org/) [29, 30]. The edge cutoff was established at 0.9 to ensure a high confidence level for obtaining dependable herb-target interactions [31]. Then, we imported the PPI data in text format into Cytoscape (http://www.cytoscape.org/) to visualize relationships and used its network analyzer plugin to calculate the degree of the PPI network. The larger a protein's degree/ betweenness centrality/ closeness centrality is, the more important the protein is in the PPI network [32].

#### 2.1.4 BisoGenet-Based Screening of PPI Network Core Targets

The BisoGenet plugin was used to perform core target screening on the constructed PPI network [33]. The screening criteria involved five parameters: degree centrality (DC), betweenness centrality (BC), closeness centrality (CC), local average connectivity (LAC), and network centrality (NC) [34-36]. By setting the parameters, the PPI network core targets were obtained. Previous reports have characterized a node as a hub if its degree exceeds twice the median degree of all nodes in the network [37-39].

#### 2.1.5 MCODE-Based Clustering Analysis of PPI Networks

For hub gene identification of the PPI network, the Molecular Complex Detection (MCODE) plug was used to identify the most important subclusters of strongly interacting nodes [40, 41]. Cluster analysis of the PPI network based on the MCODE plugin was performed, and a subset of representative terms from each cluster was selected as cluster names and converted into a networked layout. Cytoscape3.7.2 software was applied to visualize the network.

#### 2.1.6 Enrichment Analysis Based on GO and KEGG

We imported the potential targets into the metascape V3.5 database (https://metascape.org) to perform the analysis for Gene Ontology (GO) and Kyoto Encyclopedia of Genes and Genomes (KEGG) pathway enrichment and then screened for pathways with a cut-off p-value of < 0.01, a minimum count of 3, and an enrichment factor of > 1.5 [42-44].

### 2.2 Preparation of CPYJT Decoction

The CPYJT was made up of 12 herbs (Supplementary Table **1**). All herbs were purchased from the Gansu University of Chinese Medicine's Affiliated Hospital. The CPYJT aqueous extract was prepared by referring to the previous study [17]. Firstly, 10 times (V/W) water of the herb was extracted at reflux for 90 min, then 8 times (V/W) water of the herb was added for 60 min. Finally, 6 times (V/W) herb water was added for 30 min. The three filtrates were combined and concentrated under reduced pressure to the raw drug containing 5.152g/ml of Chinese medicine solution.

### 2.3 Qualitative Analysis of Components of CPYJT Water Extract

UHPLC-MS/MS analysis was performed on a UHPLC system (Thermo Fisher Scientific, Waltham, USA) with a Waters UPLC BEH C18 column (1.7 μm 2.1*100 mm). The sample injection volume was 5 μL, and the flow rate was 0.5 mL/min. Formic acid was 0.1% in water (A) and 0.1% in acetonitrile (B) in the mobile phase. Multi-step linear elution gradient program involved 0 - 11 min, 85 - 25% A; 11 - 12 min, 25 - 2% A; 12 - 14 min, 2 - 2% A; 14 - 14.1 min, 2 - 85% A. Based on the IDA acquisition mode, MS and MS/MS data were acquired using an Orbitrap Exploris 120 mass spectrometer and Xcalibur software. For each acquisition cycle, the mass range was 100 to 1500, the top four of every cycle were screened, and the corresponding MS/MS data were acquired. The following conditions were used: Sheath gas flow rate: 35 Arb, Aux gas flow rate: 15 Arb, Ion Transfer Tube Temp: 350 °C, Vaporizer Temp: 350 °C, Full ms resolution: 60000, MS/MS resolution: 15000, Collision energy: 16/32/48 in NCE mode, Spray Voltage: 5.5 kV (positive) or -4 kV(negative) [45-47].

### 2.4 Animal Experiments

Forty male SPF Sprague-Dawley (SD) rats, aged three weeks and weighing around 60±10g, were obtained from the Laboratory Animal Technology at Gansu University of Chinese Medicine. In the experimental animal center of Gansu University of Chinese Medicine, these rats were subjected to 12-hour light/dark cycles, maintained at a temperature of 23 ± 2°C, and kept at a relative humidity of 50-60%. A week of adaptive feeding was given to all rats before drug administration. Gansu University of Chinese Medicine's Animal Ethics Committee approved all procedures in accordance with the National Institutes of Health Guidelines for Laboratory Animals (NO: 2021-165).

### 2.5 Drug Administration

3,3’-Iminodipropionitrile (IDPN) was obtained from Sigma Chemical Co. (317306, Sigma, USA) and dissolved in distilled water to a concentration of 30 mg/ml. IDPN (300mg/kg) was used as a modeling drug [11, 12]. Tiapride hydrochloride tablets (Tiapride), a commonly used drug for TS treatment [48], were used as a positive control drug for CPYJT. The tiapride was purchased from Xuzhou Enhua Pharmaceutical Co., Ltd. (H32025477, Xuzhou Enhua, China) and dissolved in distilled water at 3.194 mg/ml. The intraperitoneal administration of IDPN was continued for a duration of 7 days, following which the rats were subjected to activity observation.

Activity scores were recorded on a weekly basis for a period of 1 hour and every 5 minutes. The behavioral scoring table of IDPN model rats is presented in Supplementary Table **2**. Successful modeling was demonstrated in rats exhibiting both stereotypic and locomotor behavior scores of ≥2 [49]. After successful modeling, the random numerical table was divided into three groups (n=10, each group): the model group, the Tiapride group (15ml/kg), and the CPYJT group (15ml/kg). SD rats were utilized as the blank group (n=10).

For four weeks, the blank and model groups were orally gavaged with distilled water, and the Tiapride and CPYJT groups were gavaged with the respective drugs twice a day (08:00 - 10:00 a.m., 2:00 - 4:00 p.m.). Rats were weighed daily before gavage, and the administration volume was calculated according to their body surface area. All the drugs were kept at 23°C before administering them (for alterations in rat body weight, refer to Supplementary Table **3** and Supplementary Fig. **1**). Supplementary Fig. (**1**) illustrates variations in rat behavior scores.

Four weeks later, the rats were anaesthetised and executed. Brain samples were promptly gathered and segregated for experimental purposes or rapidly frozen in liquid nitrogen, subsequently preserved at −80°C for additional research. Moreover, the experimental operators were informed only by the code.

### 2.6 Electron Microscopy [50]

Each rat's cortex, striatum, and thalamus (1×1 ×1mm3) were immersed in a solution for electron microscope fixation at 4 °C for three hours, thrice rinsed with PBS (pH 7.4), stabilized with 4% osmium acid, and then washed again with PBS. Subsequently, the samples underwent dehydration through a series of gradient alcohol washes, followed by embedding in Epon 812 resin, and finally polymerization at a temperature of 60°C for a duration of 48 hours. The samples were cut into ultrathin sections measuring 60-80 nm and stained negatively with uranyl acetate and lead citrate. The ultrastructure of mitochondria and myelin sheaths of different groups of neuronal cells was visualised using an HT7800/HT7700 electron microscope (HITACHI, Tokyo, Japan) [50].

### 2.7 Immunofluorescence

After being deparaffinized and rehydrated, the brain section slides were incubated in 0.01 M citrate buffer (pH 6.0) and subjected to high-temperature antigen retrieval for 30 min. Then, the sections were immersed in 3% H_2_O_2_-methanol solution for 10 min at 24 °C to abolish endogenous peroxidase activity. For immuno_Fluorescent labeling, sections were incubated for 2 h at 37 °C with anti-BDNF primary antibodies diluted at 1:400 (66292-1-Ig, Proteintech, China). After washing with PBS, the sections were incubated in dark for 1 h at 37 °C with FITC (111-095-003, Jackson ImmunoResearch, USA)/ TRITC(115-025-062, Jackson ImmunoResearch, USA) antibodies diluted at 1: 200. Then, nuclei were counterstained with 4′,6-diamidino-2-phenylindole (DAPI) for 5 min at room temperature. Finally, the images from the cortex, striatum, and thalamus were captured by a fluorescence microscope (Olympus VS200, Japan) at 200× magnification. Relative positive area % was analysed using ImageJ 1.53v software [51].

### 2.8 RT-PCR

Total RNA separation and extraction methods were performed according to the instructions of the Vazyme FastPure Cell/Tissue Total RNA Isolation Kit V2 (RC112, Vazyme, China). The concentration of RNA was measured by an ultraviolet spectrophotometer (NanoDrop one, USA). Reverse transcription was performed according to the instructions of the Vazyme HiScript® II Q RT SuperMix for the qPCR kit (R223, Vazyme, China). Quantitative PCR for cDNAs was conducted using the Taq Pro Universal SYBR qPCR Master Mix (Q712, Vazyme, China) and QuantStudio Real-time PCR system. Primer sequences (GenScript, China) for related genes were used (Supplementary Table. **4**). GAPDH was used as the internal control, and the data were analyzed using the 2-ΔΔCt method. The experiment was repeated three times.

### 2.9 Western Blotting

The BCA protein assay kit (A55860, Thermo Fisher Scientific, USA) was employed to measure the proteins that were isolated from the cortex, striatum, and thalamus tissue of each group. SDS-PAGE was used to separate equal proteins, and the resulting membranes (HATF00010, 0.45 μm, Millipore, USA) were then applied to them. Primary antibodies against BDNF (66292-1-Ig, Proteintech, 1:1,000), TrkB (13129-1-AP, Proteintech,1:1,000), p-TrkB (ab229908, Abcam, 1:1,000), PI3KR1 (60225-1-Ig, Proteintech, 1:2,500), p-PI3KR1 (ab182651, Abcam, 1:2,000), AKT (ab79360, Abcam, 1:2,000), p-AKT (ab38449, Abcam, 1:500), and GAPDH (TA7021, Abmart, 1:2,000) were blotted onto the membranes for an overnight period at 4 °C. Following the secondary antibody incubation, the protein bands were quantified using ImageJ 1.53v Software with the Image Lab program (Bio-Rad, USA). At a minimum, three replications of every experiment were conducted.

### 2.10 Statistical Analysis

SPSS 23.0 software (SPSS Inc. USA) was used for statistical data processing and analysis. The data corresponded to the average ± standard difference (X±SD) for the normal distribution, and multiple comparisons between groups were made using single_Factor differential analysis. Analyses of variance (ANOVA) and repeated measures tests were used for multiple group comparisons, while independent-sample t-tests were used for pairwise comparisons. Nonparametric tests were used for samples that did not follow a normal distribution. A significance level of P < 0.05 was deemed statistically significant.

## 3 RESULT

### 3.1 Network Pharmacology Studies

#### 3.1.1 Composite ingredients of CPYJT

From the 12 herbs of CPYJT: SCP, YJ, TZH, CNX, YZ, TM, JC, CT, QX, SJM, CS, SZ, a total of 252 active ingredients were retrieved from TCMSP, Symmap, ETCM, and CACS and related literature. After removing 68 no-target and duplicate ingredients, there were still 184 ingredients.

#### 3.1.2 The Construction of CPYJT-Herb-Compound-Target Network

**Fig. (1):**
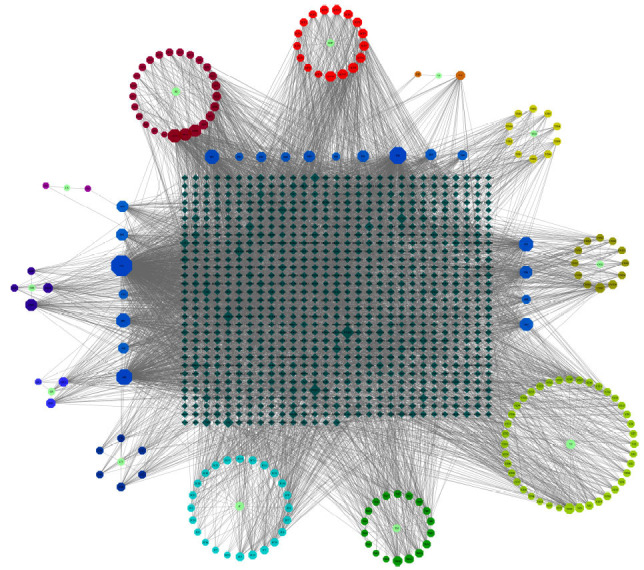
CPYJT-Drug-Component-Target Network. Cylinders: target; Circles: the 12 Chinese medicines. Eight edges: ingredients, different colors represent different corresponding ingredients; lines represent the corresponding relationship of CPYJT-herbs, herbs-chemical components, ingredients-target; the size of each node indicates the number of connections.

According to the target prediction system in TCMSP and SwissTargetPrediction, the number of putative targets for CPYJT was 5,790. Four thousand nine hundred five putative targets of the 12 herbs in CPYJT overlapped, suggesting functional potential interactions during treatment. After removing duplicated targets, the herb–ingredient–target network contained 1081 nodes (12 herbs,184 compounds, and 885 genes) and 6006 edges (Fig. **1**). This network was constructed for further network pharmacological analysis. The larger node meant more importance. According to the degree analysis, the details of top ten compounds are mentioned in Table **1**.

#### 3.1.3 Potential targets of CPYJT in Treating TS

**Fig. (2):**
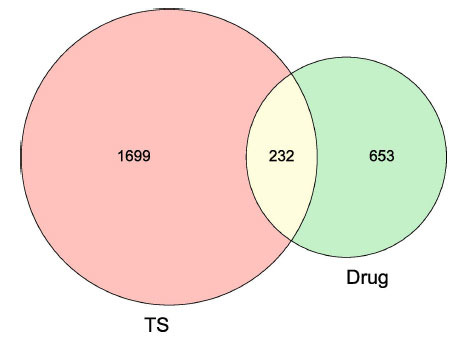
TS-CPYJT Intersection Venn Diagram. The overlapping genes were selected as core hub targets for further analysis.

In total, 2073 targets were identified for TS from the GeneCards, DrugBank, and OMIM databases. After eliminating and removing duplicate data redundancy, a total of 1932 known therapeutic targets in the treatment of TS were collected in this study. A putative CPYJT target-TS network was also constructed for further enrichment analysis. Finally, 232 bioactive compounds associated with 1932 TS-related targets were identified out of 855 CPYJT drug targets (Fig. **2**).

#### 3.1.4 PPI Network Construction and Screening of Key Active Ingredients

##### 3.1.4.1 PPI Network Construction Based on String

**Fig. (3):**
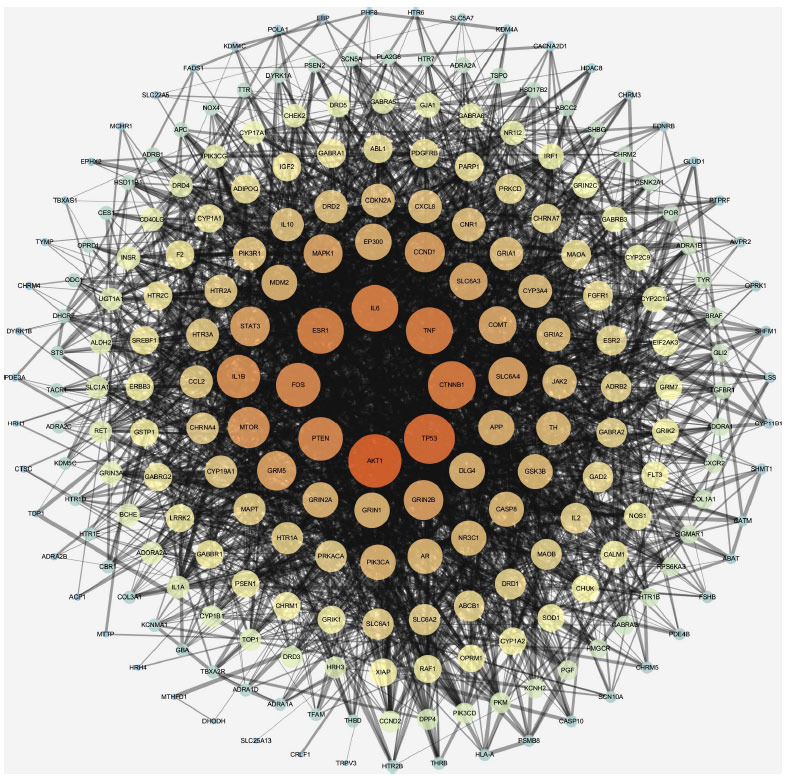
PPI network analysis diagram of CPYJT-TS intersection target. A node represents the target protein. A line represents an interaction. The node size and color represent the degree.

A total of 232 proteins were input into the STRING database, and the minimum required interaction score was set to be greater than 0.9. A total of 232 nodes and 464 edges were found in the PPI network (Fig. **3**).

##### 3.1.4.2 PPI Network Construction and Screening Based on BisoGenet

In addition, we used five different analysis parameters (DC, BC, CC, LAC, and NC) using the BisoGenet plugin to identify the core nodes in the PPI network. A high value indicates the importance of the node in the network. In the first screening, the threshold values were set at DC≥4, BC≥54.09, CC≥0.081, LAC≥1.2, and NC≥2. It resulted in 44 hub nodes and 182 edges. By setting thresholds, 160 central nodes were further improved: DC≥7, BC≥23.92, CC≥0.46, LAC≥3.27, and NC≥4.27. It resulted in 15 significant hub nodes and 58 edges. The PPI network core analysis process is shown in Fig. (**4**). We present ten core objectives in Table **2**.

#### 3.1.5 Cluster Analysis of PPI Network based on MCODE

Cluster analysis yielded ten major categories, and a “name” was assigned to each large cluster population (Table **3**). The first non-repetitive name was retained and arranged by *P*-value. The clustering results are shown in Fig. (**5**).

#### 3.1.6 GO and KEGG Pathway Analysis

To elucidate the potential mechanism and biological functions of CPYJT action in treating TS, we performed GO and KEGG pathway enrichment analyses using the clusterProfiler R package. Direct outcomes are shown in Fig. (**6**). The top 10 GO enrichment results for each category are listed in Supplementary Table **5A-C**. GO enrichment analyses significantly showed these targets correlated with multiple neurotransmitter functions and their receptor alterations. Potential targets for TS treatment were found for CPYJT through KEGG signaling pathway enrichment clustering. The top-ranking enriched pathways were related to several critical pathways associated with TS, such as the neuroactive ligand-receptor interaction, cAMP signaling pathway, and PI3K-Akt signaling pathway. Results are presented in Fig. (**7**) and Supplementary Table **6**. These findings demonstrated complex pathological features of TS and the potential of CPYJT as an anti-twitch effect.

### 3.2 Qualitative Analysis of the Major Chemical Constituents in Water Extract of CPYJT

The positive and negative ion flow diagrams of the mass spectrometry detection of the mixed extracts of the drugs in the compound formula of CPYJT are shown in Fig. (**8**). Several major classes of components of the compound were identified, including flavonoids, amino acid derivatives, phenylpropanoids, phenols, alkaloids, fatty acids, terpenoids, quinones, xanthones, isoprenol lipids, and aromatic compounds. The specific components and times are mentioned in Supplementary Table **7** and Supplementary Table **8**.

### 3.3 Morphological Changes in the Nuclear Membrane and Mitochondria

Morphological changes in the nuclear membrane and mitochondria of neuronal cells in each group are shown in Fig. (**9**). The morphology of cortical, striatal, and thalamic neuronal cells of rats in the blank group was normal. Furthermore, in the model group, compared with that of the blank group, the cytosolic boundaries of cytosolic nuclei were unclear, with the presence of indentation. Some of them showed nuclear solidification, increased electron density, increased perinuclear vacuoles, mitochondrial swelling, and mitochondrial cristae with ambiguous morphology, and severe ones showed vacuolated-like alterations. Comparing the tiapride group with the model group, the cytosolic morphology was still disrupted, with fewer perinuclear vacuole-like changes, blurred mitochondrial cristae, reduced swelling, and fewer vacuole-like changes. In the CPYJT group, the cytosolic morphology was improved, with less depression and consolidation of the cytosolic nucleus, clearer mitochondrial cristae, and reduced swelling.

### 3.4 CPYJT Acts on BDNF/TrkB/PI3K/Akt Signaling Pathway

**Fig. (10):**
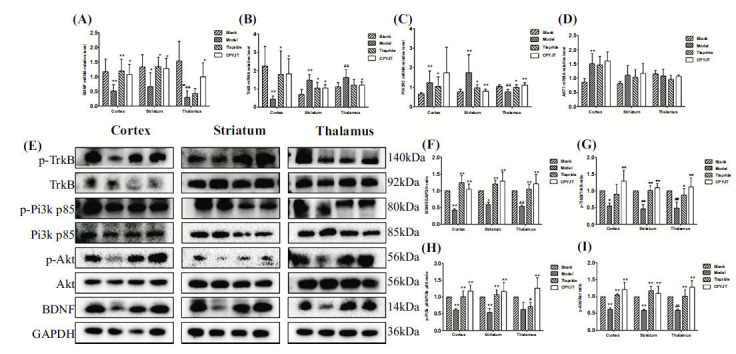
Effect of CPYJT on BDNF, TrkB, PIK3R1 and AKT. (**A-D**) Expression of BDNF, TrkB, PIK3R1, AKT mRNA relative level in the cortex, striatum, and thalamus of rats after 28 days of treatment; (**E**) Western blot analysis of BDNF, TrkB, PIK3R1, and AKT levels in cortex, striatum, and thalamus after 28 days of treatment. (**F-I**) Relative levels of BDNF, TrkB, PIK3R1, and AKT in the cortex, striatum, and thalamus after 28 days of treatment. Data are expressed as the mean±SD, n=3 for each group. ^#^*P*<0.05,^ ##^*P*<0.01, compared to the blank group; *P<0.05, ** *P*<0.01, compared to the model group.

**Fig. (11):**
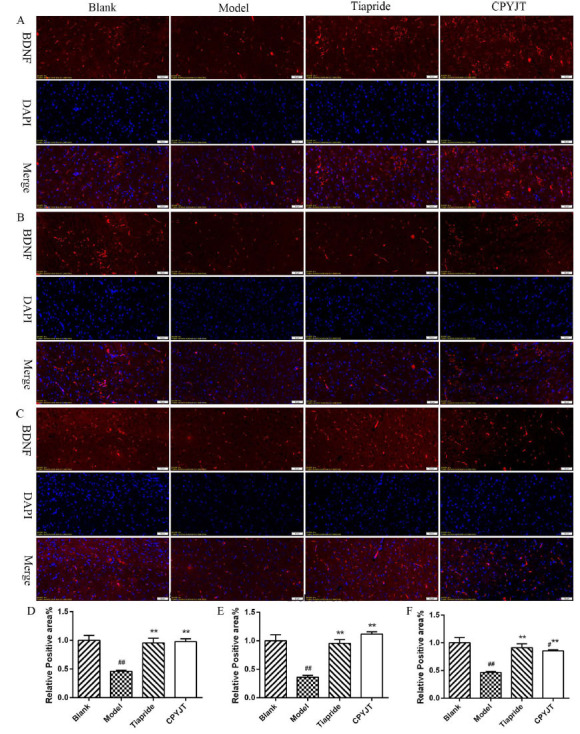
Effect of CPYJT on BDNF. **(A-C)** Representative images of BDNF—immunopositive nerve cell (red) with DAPI (blue) staining from the cortex, striatum, and thalamus; **(D_F)** Relative Quantification of BDNF+ cells in the cortex, striatum, and thalamus.

We detected the levels of BDNF, TrkB, PI3KR1, and Akt mRNA in each brain region of each group to assess the effect of CPYJT on the BDNF/TrkB/PI3K/Akt signaling pathway. BDNF mRNA levels were significantly reduced in all three brain regions in the model group, and after 28 days of administration, a significant rebound in all three brain regions was observed in the CPYJT group compared to the model group (Fig. **10A**). TrkB mRNA was significantly lower in the cortex and higher in the striatum and thalamus in the model group, and significantly higher in the cortex and lower in the striatum and thalamus in the CPYJT group after 28 days of administration (Fig. **10B**). PI3KR1 mRNA was significantly increased in the cortex and striatum and significantly decreased in the thalamus in the model group, and after 28 days of administration, it was significantly reduced in the striatum and significantly increased in the thalamus in the CPYJT group, with no significant difference in the cortex (Fig. **10C**). AKT mRNA in the model group was significantly elevated only in the cortex, and there were no significant differences in all brain regions in the CPYJT group after 28 days of administration (Fig. **10D**). We also measured phosphorylation and total levels of proteins in the BDNF/TrkB/PI3K/Akt pathway. In the model group, BDNF levels in the cortex, striatum, and thalamus were significantly reduced, and the levels of p-TrkB, p-PI3K R1, and p-AKT were reduced considerably without significant changes in the total levels. After 28 days of administration, BDNF levels in the cortex, striatum, and thalamus were significantly higher in the Tiapride and CPYJT groups. The levels of p-TrkB, p-PI3K R1, and p-AKT were significantly higher in the Tiapride and CPYJT groups compared to the model group (Fig. **10E**-**I**). BDNF immunofluorescence statistics showed a trend consistent with the previously described results. These results indicated that CPYJT elevated BDNF and promoted the phosphorylation of TrkB, PI3K, and AKT (Fig. **11A**-**F**).

## 4 DISCUSSION

Network pharmacology results showed that CPYJT treatment of TS was associated with multiple types of components and numerous biological processes. CPYJT may be able to treat the disease by modulating the abnormal neuroendocrine and neurotransmitter levels in children through various pathways. G protein-coupled receptors (GPCRs) are a general term for a large class of membrane protein receptors that bind to ligands, such as hormones, neurotransmitters, chemokines, pheromones, etc., to generate intracellular cascade reactions that ultimately exert biological effects [52, 53]. Phospholipase C-β (PLC-β) activation depends on GPCRs, which can mediate several neuroendocrine signals acting on nerve impulses and glandular secretion. The transmembrane receptors of the PLC-β pathway are mainly receptors for neurotransmitters and hormones (*e.g*., acetylcholine receptors, DAergic receptors, 5-HTergic receptors, etc.). Purkinje cells with high expression of PLC-β3 and β4 are located in the cerebellar cortex, and PLC-β4 knockdown mice develop motor dysregulation [54]. Adenylate cyclase, an effector downstream of GPCRs, is an intermediate effector molecule of intracellular protein phosphorylation, which regulates various cellular functions by modulating the synthesis of cAMP, thus participating in different pathophysiological processes in the organism. The results of cellular components showed that CPYJT mainly acted on multiple substructures of nerve cells with rich targets, which is in line with the characteristics of multi-target action of traditional Chinese medicines. This feature also poses a problem for research on the modernization of Chinese medicine. The influencing factors are complicated, and it is difficult to control the variables. The results of molecular functional analysis were analyzed in combination with KEGG pathway enrichment analysis. KEGG pathway enrichment analysis results were associated with various diseases, such as tumors, diabetes, immune system disorders, endocrine systems, etc. The main focus was on signaling pathways related to neurological disorders, e.g., neuroactive ligand-receptor interactions, cAMP signaling pathway, and multiple amino acid metabolism. The secondary results of cluster analysis of dopaminergic synapses, glutamatergic synapses, GABAergic synapses, 5-HTergic synapses, GABA biosynthesis, and retrograde endogenous cannabinoid signaling demonstrated the association of multiple signaling pathways with TS and the potential of CPYJT to exert its anti-twitching mechanism of action through multiple signaling pathways. Synthesizing the results of KEGG and GO analyses, many research directions with correlations have been found, and the commonalities would be the hotspot and future research direction of traditional Chinese medicine for the treatment of TS. Various neurotransmitters were found in the BP analysis of membrane potential and neurotransmitter regulation. CC enrichment analysis revealed major prosthetic receptor complexes, including ionotropic glutamate receptor complexes and NMDA-selective glutamate receptor complexes. The four major categories found in the MF enrichment analysis included neurotransmitter receptor activity, transmitter-gated ion channel activity, benzodiazepine receptor activity, and amino acid binding, encompassing the whole pathway related to neurotransmitter secretion, transport, receptor, and metabolism. The KEGG signaling pathway enrichment analysis identified the neurotrophic factor signaling pathway, which includes the BDNF/TrkB signaling pathway and the associated PI3K/Akt signaling pathway. Mechanistic studies on the clinical efficacy of CPYJT in the treatment of TS have been carried out previously on the content levels of monoamine neurotransmitters (DA, 5-HT, NE) in brain tissues and plasma [11, 12], the expression levels of DA transporters, the expression levels of DA receptors, and the changes of DA in the process of synaptic vesicle release, as well as the effect of blood-brain barrier permeability. Therefore, the results of the network pharmacology study on CPYJT for TS provide a theoretical basis for selecting the BDNF/TrkB/PI3K/Akt signaling pathway. The holistic and systematic nature of network pharmacology and its focus on drug-drug interactions are highly compatible with Chinese medicine theories. With Chinese medicine's understanding of the nature of the disease, network pharmacology builds a bridge between the clinical application of traditional Chinese medicine and the study of the mechanism of action of modern pharmacology. This methodology provides a new research paradigm for the transformation of TCM from an empirical to an evidence-based system of medicine, which will accelerate the discovery of TCM and improve existing drug discovery strategies. Chinese medicine and the results of research on the combination of Chinese and Western medicine have changed clinical decision-making, highlighting the leading role of Chinese medicine in the treatment of future diseases and its central role in the process of disease recovery, building a whole-process health promotion system, thus paving the way towards healthy China.

Transmission electron microscopy is an important method for studying the ultrastructure of biological samples, and various ultrastructural alterations are visible in the IDPN rat model. Previous studies have focused on structural modifications of synaptosomes in a single brain region. In contrast, there are fewer reports related to structural alterations of neuronal cells in cortical, striatal, and thalamencephalon regions with complex cellular structures. In this study, we found that the trend of structural damage in neuronal cells in the cortex, striatum, and thalamus of each brain region in the model group was the same, and the damage could be seen in terms of changes in the morphology of the nucleus pulposus, discontinuity of the nuclear membrane, and blurring of the mitochondrial cristae. Structural changes in the nucleus of neuronal cells are primarily seen in neuronal injury. A database literature search revealed no reports of ultrastructural changes in the IDPN rat model in various brain regions. Transmission electron microscopy studies in the IDPN rat model focused on morphological changes in the vestibular sensory epithelium and vestibular ganglion in cilia fusion, swelling, *etc*. [55]. The vestibular ganglion changes manifested as disruption of mitochondrial cristae and cytoplasmic vacuolization of Schwann cells, with a decrease in the number of cells [56]. Electron microscopic observation of the neuromuscular junction in the IDPN model showed the disappearance of terminal neurofilaments, decreased synaptic vesicle content, and aggregation of multilayered vesicles without degeneration, and the motor deficits and structural changes in the neural axon exhibited by the IDPN rat model represented impaired synaptic function [57]. KEGG signaling pathway enrichment analysis identified neurotrophic factors and their signaling pathways as potentially promising action pathways in the CPYJT treatment of TS. In contrast, the long-term effects of neurotrophic factors, such as BDNF, in neuronal cells are known to be protective against structural damage to neuronal cells [58] and to improve learning memory function by acting on synaptic plasticity through either long-terminal potentiation (LTP) or long-terminal depression (LTD) [59]. Therefore, the findings with CPYJT through the BDNF/TrkB/PI3K/Akt signaling pathway can be combined to examine if there is a similar trend of changes across brain regions.

Studies on the Chinese Han population support that the BDNF Val66Met polymorphism is a common genetic susceptibility for OCD and TS, suggesting that BDNF Val66Met is involved in the pathogenesis of TS [60]. In addition, BDNF polymorphisms may affect the pathophysiology of many neuropsychiatric disorders, e.g., attention deficit hyperactivity disorder (ADHD), obsessive-compulsive disorder, and movement disorders, such as TS. The val66met polymorphism of the BDNF gene has also been associated with anatomical and structural morphological variations in the hippocampus and prefrontal cortex, influencing the genetic mechanisms underlying brain morphological variations related to learning and memory [61]. BDNF promotes the formation, maturation, and stabilization of amino-acid-ergic synapses by regulating the balance of excitatory and inhibitory transmission [62, 63] and provides neural circuit formation during neuronal development. Studies have confirmed that the BDNF/TrkB signaling pathway regulates survival, morphogenesis, and synaptic plasticity in cortical, striatal, and hippocampal neurons [64]. Chronic exposure to BDNF promotes the functional maturation of GABAergic transmission in embryonic hippocampal neurons, and the current mechanism of action for the chronic effects of BDNF on the central nervous system is primarily related to learning and memory functions. In Chinese medicine studies, the compound Tang has shown clinical efficacy in children with TS. Its therapeutic effect may be associated with elevated serum BDNF levels in children [65]. The Slitrk family of proteins is highly homologous in structure to the axon guidance factor Slit and the neurotrophic factor receptor Trk. Moreover, it has a role in mediating synaptic signaling and regulating neuronal growth and development. Genetic studies have found that patients carrying the SLITRK1 variant allele exhibit TS. Slitrk1 is expressed in mature neurons capable of inducing cortical and hippocampal neuronal protrusion growth and development [66], at high levels in cortical layers III, V, and VI, and in the embryonic and postnatal brains, including the cortex, thalamus, and basal ganglia, reflecting the most common neural anatomical regions [67]. Mice with partial deletion of TrkB exhibited repetitive rotational and juvenile head-twitching episodes, and their neuronal structure revealed dendritic atrophy and loss of dendritic spines in dorsal striatal D1 medium-sized spiny neurons [68]. SLITRK5, a member of the Slitrk family, regulates the recruitment of TrkB to the postsynaptic region, which leads to BDNF-mediated axon outgrowth in striatal neurons. Genetic deletion of SLITRK5 leads to neuronal overactivity in the orbitofrontal cortex and reduced cortical striatal transmission and dendritic complexity associated with obsessive-compulsive-like behavior. However, further studies are needed to investigate its role in neural loop connections and its involvement in TS. ADHD is a common comorbidity of TS, and BDNF and TrkB expressions have also been associated with memory acquisition [69-71]. Disruption of the TrkB gene leads to impairments in learning and memory acquisition [72]. In the adult nervous system, TrkB receptors regulate synaptic strength and plasticity. Upon binding of BDNF to TrkB, tyrosine residues in the cytoplasmic structural domain of TrkB undergo autophosphorylation, which maintains LTP in hippocampal CA1 through activation of the PLC-γ1, Ras-MAPK, and PI3K/AKT signaling pathways, ultimately affecting learning [73, 74]. Activation of MAPK and PI3K among the three pathways is essential for BDNF potentiation [75, 76]. In this study, we examined the phosphorylation and total levels of mRNAs and proteins of BDNF, TrkB, PI3KR1, and Akt in the cortex, striatum, and thalamus of each group after 28 days of drug administration. The BDNF protein level in each brain region after 28 days of administration was consistent with the pattern of change of BDNF mRNA. BDNF has the function of short-term rapid promotion of neurotransmitter release and long-term regulation of synaptic structure, and synaptic structure regulation is related to the LTP and LTD effects of BDNF [77-79]. Among the short-term effects, it has been demonstrated that BDNF rapidly enhances synaptic transmission and transmitter release [80], with 30% of glutamatergic synaptic enhancement, and enhances the efficiency of neurotransmitter release through excitatory postsynaptic currents. BDNF in TS, which generally has a long disease duration, mainly affects synaptic architecture, pruning, and vulnerable neurons. Elevated BDNF protein levels in the CPYJT group and the Tiapride group were found in the cortex, striatum, and thalamus. Moreover, it is speculated that the effects of BDNF are widespread in all brain regions of the CSTC loop. In recent years, BDNF has been widely studied in TS. BDNF belongs to the category of neurotrophic factor, which has a wide range of roles in the central nervous system and affects the development of many neurological diseases. BDNF binds to the high-affinity receptor TrkB and acts on developing neurons. CPYJT affects total BDNF protein levels in various brain regions of the CSTC loop in the IDPN rat model. In this study, we found that the changes in phosphorylation and total levels of TrkB protein in cortical, striatal, and thalamic tissues of all groups after 28 days of drug administration were consistent with the changes in BDNF protein levels, which were significantly elevated in the Tiapride group except for the cortex, and in all brain regions after 28 days of drug administration in the CPYJT group. The changing trend is consistent with the upstream-downstream relationship between BDNF and TrkB in the signaling pathway. It indicates that CPYJT plays a role in the BDNF/TrkB signaling pathway. The PI3K/AKT signaling pathway is widely expressed in human tissues, is involved in cell proliferation, apoptosis, migration, and differentiation, and is closely related to nervous system regeneration. It has been demonstrated that BDNF antagonizes oxidative stress damage, improves mitochondrial function, increases protein synthesis, and promotes the differentiation of pluripotent neural stem cells by activating the PI3K/Akt signaling pathway [81]. In this study, phosphorylated and total levels of PI3KR1 protein were measured in sub-brain regions, which were consistent with the BDNF/TrkB trend and significantly decreased in all brain regions in the model group compared to the blank group. After 28 days of drug administration, they were significantly higher in all brain regions in the CPYJT group than in the model group. There was no significant difference in the elevated thalamic PI3KR1 expression levels in the Tiapride group, and the relevance was not significant in the thalamus. It is considered that the treatment cycle of the animal model can be extended to clarify whether Tiapride does not correlate with changes in PI3KR1 protein expression in thalamic brain regions. Next, in each group, the phosphorylation levels of AKT protein were detected in the cortex, striatum, and thalamus after 28 days of drug administration. The phosphorylation levels of AKT protein in the model group were found to be significantly reduced compared to the blank group. After 28 days of drug administration, these levels significantly increased in the Tiapride group and the CPYJT group compared to the model group. AKT and PI3K trends were consistent. It has been found that BDNF can play a role in changing dendritic spine morphology, increasing dendritic spine density, and promoting dendritic growth by activating the downstream PI3K/Akt pathway and Ras-MAPK pathway and increasing actin and microtubule protein polymerization [82]. Combined with the morphological changes in microtubules and neurofilaments within myelinated axons in the ultrastructure of the IDPN rat model in this study, this structural alteration may correlate with the altered expression level of BDNF in the central nervous system.

Herbal compound-based treatments can target multiple neurotransmitter systems and segments for holistic regulation, and herbal medicine can be used in conjunction with acupuncture, tuina, and other therapeutic treatments for integrated intervention [83]. Previous clinical studies [10, 13] reported that CPYJT has a significant anti-twitching effect with stable and long-lasting efficacy. Several research groups [84-86] have carried out systematic and in-depth studies on TS and have achieved certain results in verifying and exploring the targets of Chinese medicines for the recognised pathogenesis of TS. The research results have clarified the mechanism of TS treatment by traditional Chinese medicine, confirmed the reliability of the therapeutic efficacy of traditional Chinese medicine in treating TS, and also promoted the in-depth study on other pathogenesis of TS. It is crucial to enhance research on the molecular mechanism and safety of Chinese medicine therapy to enhance the efficacy and safety of therapeutic treatment for TS. Studying the mode of action of TCM involves examining three key components: chemical substances, *in vivo* processes, and mechanisms of action. This analysis reveals the relationship between material entities and life activities, which is crucial for advancing TCM. Recent advancements in histological technology have allowed for a comprehensive understanding of the processes of biological systems, including genes, mRNA, and proteins. This knowledge supports the analysis of how TCM is beneficial for living organisms. The systematic analysis of active ingredients in TCM has addressed scientific challenges in setting standards for these medicines, leading to the development of a quality standard system tailored to their complex nature [87].

## CONCLUSION

The network pharmacology results of ChangPu YuJin Tang showed that it regulates the abnormal neuroendocrine and neurotransmitter levels of TS children through multiple pathways to achieve the therapeutic purpose. Moreover, CPYJT acts through multiple neuronal cell sub-structures, along with multiple signaling pathways, which is in line with the characteristics of the compound of traditional Chinese medicine with multiple perspectives and targets. Furthermore, as validated in TS rat model experiments, CPYJT acts on the BDNF/TrkB/PI3K/AKT signaling pathway.

## AUTHORS’ CONTRIBUTIONS

M.Q. Lu mainly conducted this study. L. Gao and W.J. Gao provided technical support in network pharmacology and pharmacological studies. J. Shang contributed to the manuscript revision. Z.G. Shi and L. Gao provided the concept and ideas. All authors agree to be accountable for all aspects of work, ensuring integrity and accuracy.

## Figures and Tables

**Fig. (4):**
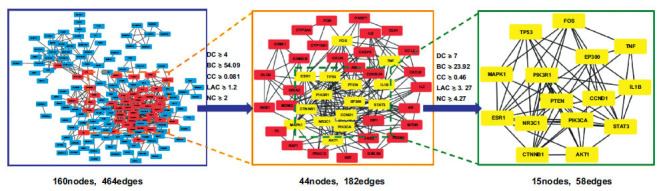
Screening of core targets for BisoGenet-based CPYJT-TS PPI network. A node represents the target protein. A line represents an interaction. Blue: primary target; pink: target with median screening once; yellow: target with median screening twice.

**Fig. (5):**
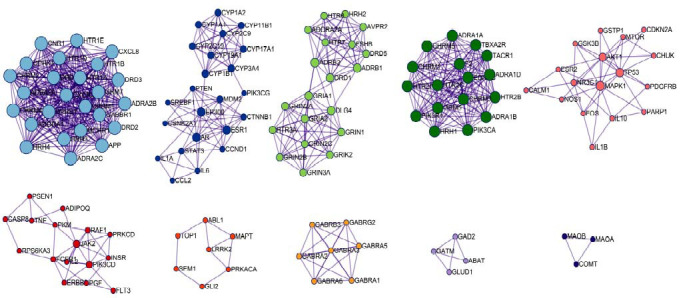
MCODE-based CPYJT-TS PPI network clustering map. A node represents the target protein, and a line represents an interaction. The different colors of the square in the diagram represent different clusters. The node size means enriched adjusted *P*-value.

**Fig. (6):**
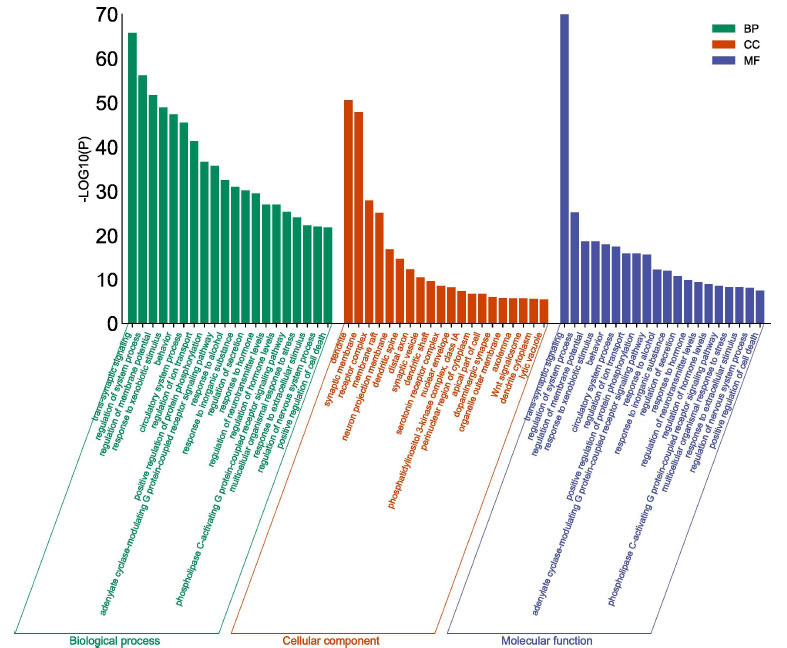
Diagram of GO enrichment analysis of CPYJT-TS Network. Each bar indicates a BP/ CC/MF term. Color means enriched type. Green: BP, orange: CC, blue: MF, and abscissa represents the number of enriched genes.

**Fig. (7):**
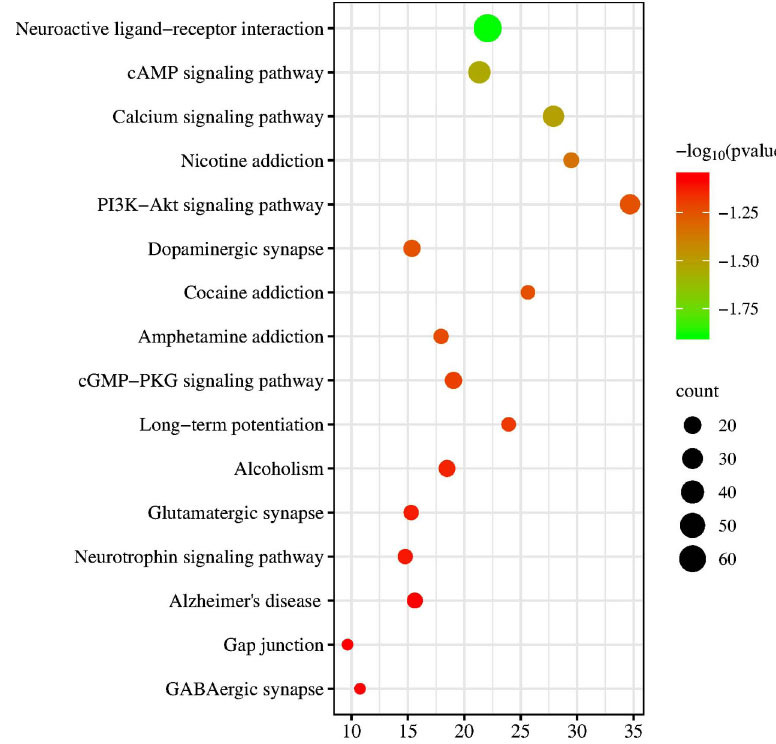
Diagram of KEGG enrichment analysis of CPYJT-TS Network. The size of each node indicates enriched counts. Abscissa represents the enriched gene ratio. Color means enriched adjusted *P*-value.

**Fig. (8):**
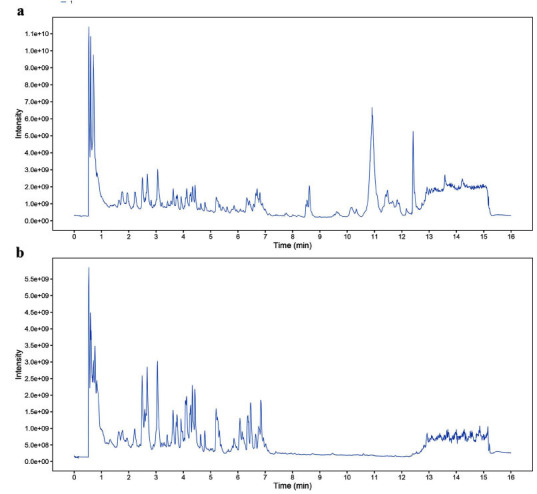
UPLC chromatograms analysis of CPYJT with the representative active ingredients: **(A)** Total ion flow chromatogram (TIC) in positive ion mode; **(B)** TIC in negative ion mode.

**Fig. (9):**
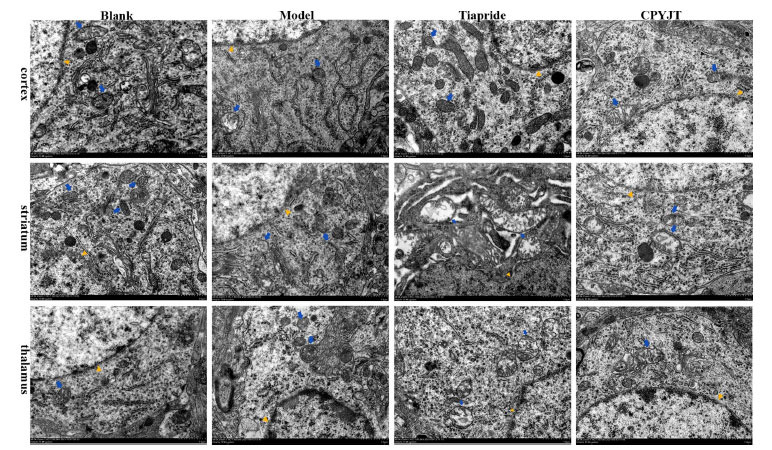
Typical images of mitochondria and nuclear membranes of neuronal cells in cortical, striatal, and thalamic brain regions of rats in each group under transmission electron microscopy (8, 000×, n=3). The orange triangles point to the nuclear membrane, and the blue arrows indicate the mitochondria.

**Table 1:** List of essential compounds of CPYJT.

**Component Name**	**CAS**	**MOL**	**Degree**
Quercetin	117-39-5	MOL000098	832
Glycine	56-40-6	MOL000050	396
Kaempferol	520-18-3	MOL000422	308
Serine	6898-95-9	MOL003969	136
Aspartic Acid	56-84-8	MOL000065	134
Beta-Sitosterol	83-46-5	MOL000358	127
Apigenin	520-36-5/8002-66-2	MOL000008	99
Glutamic Acid	56-86-0	MOL000052	86
Luteolin	491-70-3	MOL000006	77
Valine	72-18-4	MOL000067	75
Flavone	525-82-6	MOL010245	75

**Table 2:** Core targets of the BisoGenet-based CPYJT-TS PPI network.

**Name**	**DC**	**BC**	**CC**	**LAC**	**NC**
STAT3	19	130.2262661	0.5810811	6.736842105	13.4536602
PIK3R1	17	119.4153614	0.5890411	5.058823529	9.33555334
TP53	16	147.2174212	0.5657895	4.25	8.63713786
CTNNB1	16	312.5106135	0.5890411	4.125	8.88534799
AKT1	16	182.5681795	0.5890411	5.375	8.45235043
EP300	15	119.927364	0.5810811	5.466666667	8.71279831
ESR1	14	182.3709919	0.5890411	5.428571429	6.86923077
NR3C1	12	41.98395892	0.5308642	4.833333333	6.02727273
MAPK1	12	107.733757	0.5180723	3.333333333	5.67489178
CCDN1	11	45.97571828	0.5308642	4	5.07619048

**Table 3:** Cluster analysis of MCODE-based CPYJT-TS PPI network.

**Network**	**Category**	**Description**	**LOG10(P)**
MCODE 1	ko04080	Neuroactive ligand-receptor interaction	-41.2
MCODE_2	ko00140	Steroid hormone biosynthesis	-13.6
MCODE_3	ko04024	cAMP signaling pathway	-25.8
MCODE_4	hsa04020	Calcium signaling pathway	-28.0
MCODE_5	ko05215	Prostate cancer	-15.8
MCODE_6	hsa04930	Type II diabetes mellitus	-13.0
MCODE_7	ko05033	Nicotine addiction	-20.2
MCODE_8	hsa05200	Pathways in cancer	-4.0
MCODE_9	ko00250	Alanine, aspartate and glutamate metabolism	-8.2
MCODE_10	hsa00350	Tyrosine metabolism	-8.8

## Data Availability

Not applicable.

## LIST OF ABBREVIATIONS

5-HT: 5-hydroxytryptamine
ADHD: Attention Deficit Hyperactivity Disorder
BDNF: Brain-Derived Neurotrophic Factor
BC: Betweenness Centrality
CPYJT: ChangPu YuJin Tang
CC: Closeness Centrality
CSTC: Cortico-Striatal-Thalamic-Cortical
DA: Dopamine
DL: Drug-Like Properties
DC: Degree Centrality
GO: Gene Ontology
GO-BP: GO-Biological Process
GO-CC: GO-Cellular Components
GO-MF: GO-Molecular Function
GPCRs: G Protein-Coupled Receptors
PLC: Phospholipase C
KEGG: Functional Annotations and Kyoto Encyclopedia of Genes and Genomes
LAC: Local Average Connectivity
LTP: Long-Terminal Potentiation
LTD: Long-Terminal Depression
NE: Norepinephrine
NC: Network Centrality
OB: Oral Bioavailability
PPI: Protein-Protein Interaction
TS: Tourette Syndrome
TIC: Total Ion Flow Chromatogram
UHPLC-MS/MS: Ultra-High Performance Liquid Chromatography-Tandem Mass Spectrometer

## References

[1] Set K.K., Warner J.N. (2021). Tourette syndrome in children: An update.. Curr. Probl. Pediatr. Adolesc. Health Care.

[2] Deeb W., Malaty I.A., Mathews C.A. (2019). Tourette disorder and other tic disorders.. Handb. Clin. Neurol..

[3] Jafari F., Abbasi P., Rahmati M., Hodhodi T., Kazeminia M. (2022). Systematic review and meta-analysis of tourette syndrome prevalence; 1986 to 2022.. Pediatr. Neurol..

[4] Yang C., Zhang L., Zhu P., Zhu C., Guo Q. (2016). The prevalence of tic disorders for children in China.. Medicine.

[5] Eapen V., Snedden C., Črnčec R., Pick A., Sachdev P. (2016). Tourette syndrome, co-morbidities and quality of life.. Aust. N. Z. J. Psychiatry.

[6] Bloch M.H., Leckman J.F. (2009). Clinical course of Tourette syndrome.. J. Psychosom. Res..

[7] Worbe Y., Marrakchi-Kacem L., Lecomte S., Valabregue R., Poupon F., Guevara P., Tucholka A., Mangin J.F., Vidailhet M., Lehericy S., Hartmann A., Poupon C. (2015). Altered structural connectivity of cortico-striato-pallido-thalamic networks in Gilles de la Tourette syndrome.. Brain.

[8] Worbe Y., Gerardin E., Hartmann A., Valabrégue R., Chupin M., Tremblay L., Vidailhet M., Colliot O., Lehéricy S. (2010). Distinct structural changes underpin clinical phenotypes in patients with Gilles de la Tourette syndrome.. Brain.

[9] Naro A., Billeri L., Colucci V.P., Le Cause M., De Domenico C., Ciatto L., Bramanti P., Bramanti A., Calabrò R.S. (2020). Brain functional connectivity in chronic tic disorders and Gilles de la Tourette syndrome.. Prog. Neurobiol..

[10] Li Y., Shi Z., Zhao B. (2015). Clinical efficacy observation of modified Changpu Yujin Tang in the treatment of 60 cases of infantile transient tic disorder.. J. Pediat. Trad. Chinese Med..

[11] Gao H., Shi Z., Li X., Wang J., Shang J. (2016). The changes of striatal DA system in IDPN induced TS model rats and the intervention effect of Changpu Yujin Tang.. Lishizhen Med. Materia Medica Res..

[12] Feng P., Li Y., Tian W., Chen J., Shang J., Wang Q. (2023). Effects of Changpu Yujin Decoction on the expression of synaptic endocytosis related proteins in a rat model of Tourette syndrome.. Zhongchengyao.

[13] Wang N., Qin D., Xie Y., Wu X., Wang D. (2021). Hang-Yang; Li, X.; Xiong, L.; Liang, J. Traditional Chinese Medicine Strategy for Patients with Tourette Syndrome Based on Clinical Efficacy and Safety: A Meta-Analysis of 47 Randomized Controlled Trials.. BioMed Res. Int..

[14] Zheng Y., Zhang Z.J., Han X.M., Ding Y., Chen Y.Y., Wang X.F., Wei X.W., Wang M.J., Cheng Y., Nie Z.H., Zhao M., Zheng X.X. (2016). A proprietary herbal medicine (5‐ L ing G ranule) for T ourette syndrome: A randomized controlled trial.. J. Child Psychol. Psychiatry.

[15] Wang X., Hu Y., Zhou X., Li S. (2022). Editorial: Network pharmacology and traditional medicine: Setting the new standards by combining In silico and experimental work.. Front. Pharmacol..

[16] Gao H., Wang W., Shang J., Li X., Yang X., Wang J., Shi Z. (2020). Influence of ChangPu YuJin Decoction on DA Metabolic Enzyme of TS Rat Model.. Western J. Trad. Chinese Med..

[17] Gao H., Wang W., Li X., Yang X., Wang J., Shang J., Shi Z. Effect of Changpu Yujin Decoction on monoamine neurotransmitters in Tourette syndrome model rats.. Pharmacol. Clinics Chinese Materia Medica.

[18] Tiwari P., Ali S.A., Puri B., Kumar A., Datusalia A.K. (2023). Tinospora cordifolia Miers enhances the immune response in mice immunized with JEV-vaccine: A network pharmacology and experimental approach.. Phytomedicine.

[19] Saima P., Latha S., Sharma R., Kumar A. (2024). Role of network pharmacology in prediction of mechanism of neuroprotective compounds.. Methods Mol. Biol..

[20] Ru J., Li P., Wang J., Zhou W., Li B., Huang C., Li P., Guo Z., Tao W., Yang Y., Xu X., Li Y., Wang Y., Yang L. (2014). TCMSP: A database of systems pharmacology for drug discovery from herbal medicines.. J. Cheminform..

[21] Yan P., Wei Y., Wang M., Tao J., Ouyang H., Du Z., Li S., Jiang H. (2022). Network pharmacology combined with metabolomics and lipidomics to reveal the hypolipidemic mechanism of Alismatis rhizoma in hyperlipidemic mice.. Food Funct..

[22] Xu X., Zhang W., Huang C., Li Y., Yu H., Wang Y., Duan J., Ling Y. (2012). A novel chemometric method for the prediction of human oral bioavailability.. Int. J. Mol. Sci..

[23] Yu H., Chen J., Xu X., Li Y., Zhao H., Fang Y., Li X., Zhou W., Wang W., Wang Y. (2012). A systematic prediction of multiple drug-target interactions from chemical, genomic, and pharmacological data.. PLoS One.

[24] Daina A., Michielin O., Zoete V. (2017). SwissADME: A free web tool to evaluate pharmacokinetics, drug-likeness and medicinal chemistry friendliness of small molecules.. Sci. Rep..

[25] Daina A., Zoete V. (2016). A BOILED‐Egg to predict gastrointestinal absorption and brain penetration of small molecules.. ChemMedChem.

[26] Rappaport N., Twik M., Plaschkes I., Nudel R., Iny Stein T., Levitt J., Gershoni M., Morrey C.P., Safran M., Lancet D. (2017). MalaCards: An amalgamated human disease compendium with diverse clinical and genetic annotation and structured search.. Nucleic Acids Res..

[27] Wishart D.S., Feunang Y.D., Guo A.C., Lo E.J., Marcu A., Grant J.R., Sajed T., Johnson D., Li C., Sayeeda Z., Assempour N., Iynkkaran I., Liu Y., Maciejewski A., Gale N., Wilson A., Chin L., Cummings R., Le D., Pon A., Knox C., Wilson M. (2018). DrugBank 5.0: A major update to the DrugBank database for 2018.. Nucleic Acids Res..

[28] Yuan N., Gong L., Tang K., He L., Hao W., Li X., Ma Q., Chen J. (2020). An integrated pharmacology-based analysis for antidepressant mechanism of chinese herbal formula Xiao-Yao-San.. Front. Pharmacol..

[29] Szklarczyk D., Gable A.L., Lyon D., Junge A., Wyder S., Huerta-Cepas J., Simonovic M., Doncheva N.T., Morris J.H., Bork P., Jensen L.J., Mering C. (2019). STRING v11: protein–protein association networks with increased coverage, supporting functional discovery in genome-wide experimental datasets.. Nucleic Acids Res..

[30] Zhang J.Y., Hong C.L., Chen H.S., Zhou X.J., Zhang Y.J., Efferth T., Yang Y.X., Li C.Y. (2019). Target identification of active constituents of shen qi wan to treat kidney yang deficiency using computational target fishing and network pharmacology.. Front. Pharmacol..

[31] Szklarczyk D., Gable A.L., Nastou K.C., Lyon D., Kirsch R., Pyysalo S., Doncheva N.T., Legeay M., Fang T., Bork P., Jensen L.J., von Mering C. (2021). The STRING database in 2021: Customizable protein–protein networks, and functional characterization of user-uploaded gene/measurement sets.. Nucleic Acids Res..

[32] Valente T.W., Fujimoto K. (2010). Bridging: Locating critical connectors in a network.. Soc. Networks.

[33] Yu G., Wang W., Wang X., Xu M., Zhang L., Ding L., Guo R., Shi Y. (2018). Network pharmacology-based strategy to investigate pharmacological mechanisms of Zuojinwan for treatment of gastritis.. BMC Complement. Altern. Med..

[34] Missiuro P.V., Liu K., Zou L., Ross B.C., Zhao G., Liu J.S., Ge H. (2009). Information flow analysis of interactome networks.. PLOS Comput. Biol..

[35] Tang Y., Li M., Wang J., Pan Y., Wu F.X. (2015). CytoNCA: A cytoscape plugin for centrality analysis and evaluation of protein interaction networks.. Biosystems.

[36] Raman K., Damaraju N., Joshi G.K. (2014). The organisational structure of protein networks: Revisiting the centrality–lethality hypothesis.. Syst. Synth. Biol..

[37] Guo Q., Zhong M., Xu H., Mao X., Zhang Y., Lin N. (2015). A systems biology perspective on the molecular mechanisms underlying the therapeutic effects of buyang huanwu decoction on ischemic stroke.. Rejuvenation Res..

[38] Zhang Y., Guo X., Wang D., Li R., Li X., Xu Y., Liu Z., Song Z., Lin Y., Li Z., Lin N. (2014). A systems biology-based investigation into the therapeutic effects of Gansui Banxia Tang on reversing the imbalanced network of hepatocellular carcinoma.. Sci. Rep..

[39] Zhang Y., Wang D., Tan S., Xu H., Liu C., Lin N. (2013). A systems biology-based investigation into the pharmacological mechanisms of wu tou tang acting on rheumatoid arthritis by integrating network analysis.. Evid. Based Complement. Alternat. Med..

[40] Bader G.D., Hogue C.W.V. (2003). An automated method for finding molecular complexes in large protein interaction networks.. BMC Bioinformatics.

[41] Shannon P., Markiel A., Ozier O., Baliga N.S., Wang J.T., Ramage D., Amin N., Schwikowski B., Ideker T. (2003). Cytoscape: A software environment for integrated models of biomolecular interaction networks.. Genome Res..

[42] Zhou Y., Zhou B., Pache L., Chang M., Khodabakhshi A.H., Tanaseichuk O., Benner C., Chanda S.K. (2019). Metascape provides a biologist-oriented resource for the analysis of systems-level datasets.. Nat. Commun..

[43] Guo Y., Gan H., Xu S., Zeng G., Xiao L., Ding Z., Zhu J., Xiong X., Fu Z. (2023). Deciphering the mechanism of xijiao dihuang decoction in treating psoriasis by network pharmacology and experimental validation.. Drug Des. Devel. Ther..

[44] Pei Z., Guo X., Zheng F., Yang Z., Li T., Yu Z., Li X., Guo X., Chen Q., Fu C., Tang T., Feng D., Wang Y. (2024). Xuefu Zhuyu decoction promotes synaptic plasticity by targeting miR-191a-5p/BDNF-TrkB axis in severe traumatic brain injury.. Phytomedicine.

[45] De Vos R.C.H., Moco S., Lommen A., Keurentjes J.J.B., Bino R.J., Hall R.D. (2007). Untargeted large-scale plant metabolomics using liquid chromatography coupled to mass spectrometry.. Nat. Protoc..

[46] Doppler M., Kluger B., Bueschl C., Schneider C., Krska R., Delcambre S., Hiller K., Lemmens M., Schuhmacher R. (2016). Stable isotope-assisted evaluation of different extraction solvents for untargeted metabolomics of plants.. Int. J. Mol. Sci..

[47] Liu Y., Hu Y., Qin X. (2018). Metabonomics study on interventions of Huangqi Jianzhong Decoction against chronic atrophic gastritis in rats.. Chin. Tradit. Herbal Drugs.

[48] Fekete S., Egberts K., Preissler T., Wewetzer C., Mehler-Wex C., Romanos M., Gerlach M. (2021). Estimation of a preliminary therapeutic reference range for children and adolescents with tic disorders treated with tiapride.. Eur. J. Clin. Pharmacol..

[49] Cavanna A.E., Eddy C., Rickards H.E. (2019). Cognitive functioning in Tourette syndrome.. Discov. Med..

[50] Yuan H., Ni X., Zheng M., Han X., Song Y., Yu M. (2019). Effect of catalpol on behavior and neurodevelopment in an ADHD rat model.. Biomed. Pharmacother..

[51] Song Y., Yuan H., Chen T., Lu M., Lei S., Han X. (2021). An Shen Ding Zhi Ling alleviates symptoms of attention deficit hyperactivity disorder *via* anti-inflammatory effects in spontaneous hypertensive rats.. Front. Pharmacol..

[52] Bondarev A.D., Attwood M.M., Jonsson J., Chubarev V.N., Tarasov V.V., Schiöth H.B. (2020). Opportunities and challenges for drug discovery in modulating Adhesion G protein-coupled receptor (GPCR) functions.. Expert Opin. Drug Discov..

[53] Singh R., Kumar A., Lather V., Sharma R., Pandita D. (2024). Identification of novel signal of Raynaud’s phenomenon with Calcitonin Gene-Related Peptide(CGRP) antagonists using data mining algorithms and network pharmacological approaches.. Expert Opin. Drug Saf..

[54] Rusciano I., Marvi M.V., Owusu Obeng E., Mongiorgi S., Ramazzotti G., Follo M.Y., Zoli M., Morandi L., Asioli S., Fabbri V.P., McCubrey J.A., Suh P.G., Manzoli L., Cocco L., Ratti S. (2021). Location-dependent role of phospholipase C signaling in the brain: Physiology and pathology.. Adv. Biol. Regul..

[55] Llorens J., Demêmes D., Sans A. (1993). The behavioral syndrome caused by 3,3′-iminodipropionitrile and related nitriles in the rat is associated with degeneration of the vestibular sensory hair cells.. Toxicol. Appl. Pharmacol..

[56] Ikenouchi-Sugita A., Yoshimura R., Hayashi K., Ueda N., Umene-Nakano W., Hori H., Nakamura J. (2009). A case of late-onset Tourette’s disorder successfully treated with aripiprazole: View from blood levels of catecholamine metabolites and brain-derived neurotrophic factor (BDNF).. World J. Biol. Psychiatry,.

[57] Wang Z., Maia T.V., Marsh R., Colibazzi T., Gerber A., Peterson B.S. (2011). The neural circuits that generate tics in Tourette’s syndrome.. Am. J. Psychiatry.

[58] McCairn K.W., Bronfeld M., Belelovsky K., Bar-Gad I. (2009). The neurophysiological correlates of motor tics following focal striatal disinhibition.. Brain.

[59] Worbe Y., Baup N., Grabli D., Chaigneau M., Mounayar S., McCairn K., Féger J., Tremblay L. (2009). Behavioral and movement disorders induced by local inhibitory dysfunction in primate striatum.. Cereb. Cortex.

[60] Liu S., Cui J., Niu Z., Yi M., Zhang X., Che F., Ma X. (2015). Do obsessive–compulsive disorder and Tourette syndrome share a common susceptibility gene? An association study of the BDNF Val66Met polymorphism in the Chinese Han population.. World J. Biol. Psychiatry.

[61] Shang Y., Wang N., Zhang E., Liu Q., Li H., Zhao X. (2022). The brain-derived neurotrophic factor Val66Met polymorphism is associated with female obsessive-compulsive disorder: An updated meta-analysis of 2765 obsessive-compulsive disorder cases and 5558 controls.. Front. Psychiatry.

[62] Kuhn J., Janouschek H., Raptis M., Rex S., Lenartz D., Neuner I., Mottaghy F.M., Schneider F., Schaefer W.M., Sturm V., Gründer G., Vernaleken I. (2012). *In vivo* evidence of deep brain stimulation-induced dopaminergic modulation in Tourette’s syndrome.. Biol. Psychiatry.

[63] Lai K.N., Shute J.K., Lindley I.J., Lai F.M., Yu A.W.Y., Li P.K.T., Lai C.K.W. (1996). Neutrophil attractant protein-1 interleukin 8 and its autoantibodies in IgA nephropathy.. Clin. Immunol. Immunopathol..

[64] Kuo H., Liu F. (2019). Synaptic wiring of corticostriatal circuits in basal ganglia: Insights into the pathogenesis of neuropsychiatric disorders.. eneuro.

[65] Yu W., Zhang X., Shi X., Niu Y., Cui X. (2019). Effects of jianpizhidong decoction on expression of serum BDNF in Tourette syndrom children.. Zhonghua Zhongyiyao Xuekan.

[66] Won S.Y., Lee P., Kim H.M. (2019). Synaptic organizer: Slitrks and type IIa receptor protein tyrosine phosphatases.. Curr. Opin. Struct. Biol..

[67] Paschou P. (2013). The genetic basis of Gilles de la Tourette Syndrome.. Neurosci. Biobehav. Rev..

[68] Engeln M., Song Y., Chandra R., La A., Fox M.E., Evans B., Turner M.D., Thomas S., Francis T.C., Hertzano R., Lobo M.K. (2021). Individual differences in stereotypy and neuron subtype translatome with TrkB deletion.. Mol. Psychiatry.

[69] Ma Y.L., Wang H.L., Wu H.C., Wei C.L., Lee E.H.Y. (1997). Brain-derived neurotrophic factor antisense oligonucleotide impairs memory retention and inhibits long-term potentiation in rats.. Neuroscience.

[70] Mizuno M., Yamada K., Olariu A., Nawa H., Nabeshima T. (2000). Involvement of brain-derived neurotrophic factor in spatial memory formation and maintenance in a radial arm maze test in rats.. J. Neurosci..

[71] Yamada K., Mizuno M., Nabeshima T. (2002). Role for brain-derived neurotrophic factor in learning and memory.. Life Sci..

[72] Minichiello L., Korte M., Wolfer D., Kühn R., Unsicker K., Cestari V., Rossi-Arnaud C., Lipp H.P., Bonhoeffer T., Klein R. (1999). Essential role for TrkB receptors in hippocampus-mediated learning.. Neuron.

[73] Baydyuk M., Russell T., Liao G.Y., Zang K., An J.J., Reichardt L.F., Xu B. (2011). TrkB receptor controls striatal formation by regulating the number of newborn striatal neurons.. Proc. Natl. Acad. Sci. USA.

[74] Huang E.J., Reichardt L.F. (2003). Trk receptors: Roles in neuronal signal transduction.. Annu. Rev. Biochem..

[75] Lu B., Gottschalk W. (2000). Modulation of hippocampal synaptic transmission and plasticity by neurotrophins.. Prog. Brain Res..

[76] Bhati V., Kumar A., Lather V., Sharma R., Pandita D. (2023). Association of temozolomide with progressive multifocal leukoencephalopathy: A disproportionality analysis integrated with network pharmacology.. Expert Opin. Drug Saf..

[77] Krivokhizh V.N., Bertash V.I. (1980). Clinical significance of leukocyte cytochemical indices in tuberculosis in children. Probl. Tuberk..

[78] Kang H., Welcher A.A., Shelton D., Schuman E.M. (1997). Neurotrophins and time: Different roles for TrkB signaling in hippocampal long-term potentiation.. Neuron.

[79] Liu J.H., Zhang M., Wang Q., Wu D.Y., Jie W., Hu N.Y., Lan J.Z., Zeng K., Li S.J., Li X.W., Yang J.M., Gao T.M. (2022). Distinct roles of astroglia and neurons in synaptic plasticity and memory.. Mol. Psychiatry.

[80] Xu B., Zang K., Ruff N.L., Zhang Y.A., McConnell S.K., Stryker M.P., Reichardt L.F. (2000). Cortical degeneration in the absence of neurotrophin signaling: Dendritic retraction and neuronal loss after removal of the receptor TrkB.. Neuron.

[81] Daftary S.S., Calderon G., Rios M. (2012). Essential role of brain-derived neurotrophic factor in the regulation of serotonin transmission in the basolateral amygdala.. Neuroscience.

[82] (2018). von Bohlen und Halbach, O.; von Bohlen und Halbach, V. BDNF effects on dendritic spine morphology and hippocampal function.. Cell Tissue Res..

[83] Lin J., Guo H., Qin H., Zhang X., Sheng J. (2024). Integration of meta-analysis and network pharmacology analysis to investigate the pharmacological mechanisms of traditional Chinese medicine in the treatment of hepatocellular carcinoma.. Front. Pharmacol..

[84] Long H., Wang C., Ruan J., Zhang M., Huang Y. (2019). Gastrodin attenuates neuroinflammation in DOI‐induce Tourette syndrome in rats.. J. Biochem. Mol. Toxicol..

[85] Zhang W., Yu W., Liu X., Wang Q., Bai X., Cui X., Wang S. (2020). Effect of Jian-Pi-Zhi-Dong decoction on the amino acid neurotransmitters in a rat model of tourette syndrome and comorbid anxiety disorder.. Front. Psychiatry.

[86] Zhang F., Li A. (2015). Dual ameliorative effects of Ningdong granule on dopamine in rat models of Tourette’s syndrome.. Sci. Rep..

[87] Yang W., Zhang Y., Wu W., Huang L., Guo D., Liu C. (2017). Approaches to establish Q-markers for the quality standards of traditional Chinese medicines.. Acta Pharm. Sin. B.

