# Supplementary material for: Network Pharmacology Combined with Animal Models to Investigate the Mechanism of ChangPu YuJin Tang in the Treatment of Tourette Syndrome
Source: Comb Chem High Throughput Screen. 2024 May 3;28(1):166–84. doi: 10.2174/0113862073295447240430113053 (PMC11826910; doi:10.2174/0113862073295447240430113053)
Supplement: Supplementary file 1 [file CCHTS-28-1-166_SD1.pdf]

# Supplementary Material

# Network Pharmacology Combined with Animal Models to Investigate the Mechanism of ChangPu YuJin Tang in the Treatment of Tourette Syndrome

Man-Qi Lu[1], Zheng-Gang Shi[1,*], Jing Shang[1], Lei Gao[1], Wei-Jiao Gao[1] and Lü Gao[2]

[1]*Gansu University Of Chinese Medicine Clinical College of Chinese Medicine, Lanzhou, 730000, China;* [2]*Shanxi University Of Chinese Medicine Third Clinical Medical College Pediatric Teaching and Research Department, Taiyuan 140100, China*

**Supplementary Table 1. Composition of CPYJT.**

| Component | Chinese name | Family | Part used | Origin (PR China)/Batch number | Weight (g) |
|---|---|---|---|---|---|
| Acortw *tatarinowii* Schott | Shi Chang Pu | Acoraceae | Rhizome | An Hui/202012003 | 10 |
| *Curcuma zvenyujin* Y. H_ Chen et C. Ling | Yu Jin | Zingiberaceae | Roots | Guang Xi/210225 | 10 |
| *Polygala tenuifolia* Willd. | Yuan Zhi | Polygalaceae | Roots | Shan Xi/20210302 | 8 |
| *Cyathula officinalis* Kuan | Chuan Niu Xi | Amaranthaceae | Roots | Si Chuan/20090201 | 10 |
| *Bambusa textilis* McClure | Tian Zhu Huang | Poaceae | Dried lumps of stem endocrine | Si Chuan/20190903 | 8 |
| Gastrodia elata BL. | Tian Ma | Orchidaceae | dry tuber | Shan Xi/21020704 | 8 |
| Dry body of 4th-5th instar larvae of *Bombyx mori* Linnaeus ( or artificially inoculated ) by *Beauveria bassiana* （Bals. ）Vuilknt | Jiang Can | Bombycidae | Dried body | Si Chuan/20190617 | 8 |
| *Cryptotympana pustulata* Fabriciu | Chan Tui | Cicadidae | Falling shell | Shan Dong/D2101062 | 8 |
| *Buthus martensii* Karsch | Quan Xie | Buthidae | Dried body | Gan Su/20111601 | 3 |
| MAGNETITUM | Ci Shi | oxide materials | Mineral matter | Shan Dong20112802 | 20 |
| *Haliotis diversicolor* Reeve | Shi Jue Ming | haliotidae | Oyster shell | Zhe Jiang/200912 | 20 |
| *Crataegus pinnatifida* Bge. | Jiao Shan Zha | Rosaceae | Dry ripe fruit | Shan Dong/201022 | 8 |

**Supplementary Table 2A. Results of GO-biological process enrichment analysis of CPYJT-TS Network.**

| Category | Description | -LOG10（P） |
|---|---|---|
| GO:0099537 | trans-synaptic signaling | 65.76 |
| GO:0044057 | regulation of system process | 56.23 |
| GO:0042391 | regulation of membrane potential | 51.78 |
| GO:0009410 | response to xenobiotic stimulus | 48.97 |

| | | |
|---|---|---|
| GO:0007610 | behavior | 47.43 |
| GO:0003013 | circulatory system process | 45.51 |
| GO:0043269 | regulation of ion transport | 41.37 |
| GO:0001934 | positive regulation of protein phosphorylation | 36.67 |
| GO:0007188 | adenylate cyclase-modulating G protein-coupled receptor signaling pathway | 35.77 |
| GO:0097305 | response to alcohol | 32.56 |
| GO:0010035 | response to inorganic substance | 31.02 |
| GO:0051046 | regulation of secretion | 30.22 |
| GO:0009725 | response to hormone | 29.58 |
| GO:0001505 | regulation of neurotransmitter levels | 27.05 |
| GO:0010817 | regulation of hormone levels | 27.03 |
| GO:0007200 | phospholipase C-activating G protein-coupled receptor signaling pathway | 25.43 |
| GO:0033555 | multicellular organismal response to stress | 24.12 |
| GO:0009991 | response to extracellular stimulus | 22.36 |
| GO:0031644 | regulation of nervous system process | 22.04 |
| GO:0010942 | positive regulation of cell death | 21.85 |

**Supplementary Table 2B. Results of GO- Cellular Components enrichment analysis of CPYJT-TS Network.**

| Category | Description | -LOG10（P） |
|---|---|---|
| GO:0030425 | dendrite | 50.57 |
| GO:0097060 | synaptic membrane | 47.89 |
| GO:0043235 | receptor complex | 27.94 |
| GO:0045121 | membrane raft | 25.14 |
| GO:0032589 | neuron projection membrane | 16.9 |
| GO:0043197 | dendritic spine | 14.77 |
| GO:0150034 | distal axon | 12.41 |
| GO:0008021 | synaptic vesicle | 10.42 |
| GO:0043198 | dendritic shaft | 9.57 |
| GO:0098665 | serotonin receptor complex | 8.47 |
| GO:0005635 | nuclear envelope | 8.17 |
| GO:0005943 | phosphatidylinositol 3-kinase complex, class IA | 7.3 |
| GO:0048471 | perinuclear region of cytoplasm | 6.7 |
| GO:0045177 | apical part of cell | 6.67 |
| GO:0098691 | dopaminergic synapse | 5.97 |
| GO:0031968 | organelle outer membrane | 5.7 |
| GO:0030673 | axolemma | 5.64 |
| GO:1990909 | Wnt signalosome | 5.64 |
| GO:0032839 | dendrite cytoplasm | 5.51 |
| GO:0000323 | lytic vacuole | 5.41 |

**Supplementary Table 2C. Results of GO-molecular functional enrichment analysis of CPYJT-TS Network.**

| Category | Description | -LOG10（P ） |
|---|---|---|
| GO:0030594 | neurotransmitter receptor activity | 69.99 |
| GO:0022824 | transmitter-gated ion channel activity | 25.31 |
| GO:0043176 | amine binding | 18.78 |
| GO:1901338 | catecholamine binding | 18.78 |
| GO:0099528 | G protein-coupled neurotransmitter receptor activity | 18.1 |
| GO:0019901 | protein kinase binding | 17.51 |
| GO:0004672 | protein kinase activity | 16.03 |
| GO:0004935 | adrenergic receptor activity | 16.02 |
| GO:0016705 | oxidoreductase activity, acting on paired donors, with incorporation or reduction of molecular oxygen | 15.78 |
| GO:0008503 | benzodiazepine receptor activity | 12.33 |
| GO:0019904 | protein domain specific binding | 12.12 |
| GO:0016597 | amino acid binding | 10.81 |
| GO:0004969 | histamine receptor activity | 9.82 |
| GO:0042277 | peptide binding | 9.31 |
| GO:0042562 | hormone binding | 8.85 |
| GO:0005126 | cytokine receptor binding | 8.46 |
| GO:0004879 | nuclear receptor activity | 8.22 |
| GO:0043560 | insulin receptor substrate binding | 8.2 |
| GO:0004713 | protein tyrosine kinase activity | 8.04 |
| GO:0019902 | phosphatase binding | 7.44 |

**Supplementary Table 3. Results of KEGG enrichment analysis of CPYJT-TS Network.**

| Category | Description | -LOG10（P） |
|---|---|---|
| hsa04080 | Neuroactive ligand-receptor interaction | 81.17243 |
| ko04024 | cAMP signaling pathway | 34.92922 |
| hsa04020 | Calcium signaling pathway | 32.4666 |
| ko05033 | Nicotine addiction | 22.4347 |
| hsa04151 | PI3K-Akt signaling pathway | 17.85952 |
| ko04728 | Dopaminergic synapse | 17.7939 |
| ko05030 | Cocaine addiction | 17.63119 |
| ko05031 | Amphetamine addiction | 16.9813 |
| ko04022 | cGMP-PKG signaling pathway | 15.90431 |
| hsa04720 | Long-term potentiation | 15.52676 |
| ko05034 | Alcoholism | 13.90851 |
| ko04724 | Glutamatergic synapse | 13.45436 |
| hsa04722 | Neurotrophin signaling pathway | 13.17214 |

| | | |
|---|---|---|
| hsa05010 | Alzheimer's disease | 11.96965 |
| ko04727 | GABAergic synapse | 11.06071 |
| hsa04540 | Gap junction | 11.06071 |

**Supplementary Table 4. Negative ion mode composition table**

| Name | Formula | mzmed | rtmed | ppm | ms2Adduct | Class |
|---|---|---|---|---|---|---|
| 3-(3,4-dihydroxyphenyl）-5,7-dihydroxy-6,8-bis(3-methylbut-2-enyl）chromen-4-one | C25H26O6 | 421.1651989 | 579.627 | 1.90214943955954 | [M-H]- | flavonoids |
| 5-OXO-D-PROLINE | C5H7NO3 | 128.035501 | 423.545 | 3.89743429853817 | [M-H]- | Amino acid derivatives |
| Bavachalcone | C20H20O4 | 337.1450217 | 442.401 | 0.0644105107131636 | [M+CH2-H] | flavonoids |
| Curcumenol | C15H22O2 | 233.1547355 | 546.618 | 1.13448997917715 | [M-H]- | terpenoids |
| Isokobusone | C14H22O2 | 221.1544415 | 403.885 | 2.52517821524022 | [M-H]- | terpenoids |
| Isopeonol | C9H10O3 | 165.0559925 | 179.842 | 0.0455611572002131 | [M-H]- | Phenols |
| Oleanolic acid | C30H48O3 | 455.3539378 | 694.325 | 2.05951380923674 | [M-H]- | terpenoids |
| p-Hydroxy-cinnamic acid | C9H8O3 | 163.0403924 | 238.74 | 2.40654515317916 | [M-H] | phenylpropanoids |
| p-Hydroxybenzaldehyde | C7H6O2 | 121.0298524 | 78.4181 | 1.21978217038932 | [M-H] | Phenols |
| Phenylalanine | C9H11NO2 | 164.0719077 | 94.631 | 0.562442488080911 | [M-H]- | Amino acid derivatives |
| Protocatechuic acid | C7H6O4 | 153.0195857 | 41.3247 | 2.70723498559934 | [M-H] | Phenols |
| Sumaresinolic acid | C30H48O4 | 471.3472883 | 535.913 | 1.50992671795771 | [M-H]- | terpenoids |
| 2,3-Dihydroxybenzoic acid | C7H6O4 | 153.019609 | 105.679 | 2.55520406540734 | [M-H]- | |
| L-ASPARTATE | C4H7NO4 | 132.0304314 | 74.7872 | 3.26740088961421 | [M-H]- | Amino acid derivatives |
| Methyl hexadecanoate | C17H34O2 | 315.2544421 | 524.494 | 1.40233835270653 | [M+HCOO] | Aliphatic acyl |
| Asiatic Acid | C30H48O5 | 487.3449071 | 446.171 | 1.86137601669645 | [M-H]- | terpenoids |
| Protocatechualdehyde | C7H6O3 | 137.0244674 | 43.3027 | 3.88673432572438 | [M-H] | Phenols |
| FA 18:1+3O | C18H34O5 | 329.233506 | 403.5715 | 1.50038512469124 | [M-H]- | |
| Quercetin-3-O-galactoside | C21H20O12 | 463.0877761 | 99.9227 | 2.64298869520447 | [M-H]- | flavonoids |
| Vanillic acid | C8H8O4 | 167.0347302 | 238.74 | 1.61549700313491 | [M-H]- | Organic acids and derivatives |
| Isoliquiritigenin | C15H12O4 | 255.0664755 | 192.535 | 2.05645424806492 | [M-H] | flavonoids |
| Gentisic acid | C7H6O4 | 153.0196913 | 917.162 | 2.01730778813025 | [M-H] | Xanthones |
| Carpachromene | C20H16O5 | 335.0933872 | 465.698 | 1.15552485889466 | [M-H]- | flavonoids |
| Soyasapogenol E base + O-HexA-Hex-dHex | C48H76O18 | 939.4998339 | 440.445 | 1.95198740517493 | [M-H]- | terpenoids |

| | | | | | | |
|---|---|---|---|---|---|---|
| Nicotinic acid | C6H5NO2 | 122.0248777 | 316.22 | 1.00236232422103 | [M-H] | alkaloid |
| Methyl 4-hydroxycinnamate | C10H10O3 | 177.0559245 | 87.9407 | 0.426677199605737 | [M-H] | phenylpro-panoids |
| rutamarin | C21H24O5 | 355.1580886 | 776.425 | 0.249521601294229 | [M-H]- | phenylpro-panoids |
| Genistein | C15H10O5 | 269.0459133 | 451.845 | 0.322210933975326 | [M-H]- | flavonoids |
| Citric acid | C6H8O7 | 191.0197917 | 42.7877 | 1.09025385864807 | [M-H] | Carboxylic acids and derivatives |
| Licoricesaponin G2 | C42H62O17 | 837.3916976 | 326.201 | 0.361179761982685 | [M-H]- | terpenoids |
| Azuleno(5,6-c)furan-1(3H)-one, 4,4a,5,6,7,7a,8,9-octahydro-3,4,8-trihydroxy-6,6,8-trimethyl- | C15H22O5 | 281.1397216 | 237.648 | 2.56666371041515 | [M-H]- | terpenoids |
| Salicylic acid | C7H6O3 | 137.0244712 | 158.951 | 3.43902816609197 | [M-H]- | Phenols |
| 3-hydroxybenzoic acid | C7H6O3 | 137.0248349 | 68.1623 | 1.20484458258541 | [M-H]- | Aromatici-ty |
| Enoxolone | C30H46O4 | 469.332487 | 601.533 | 1.09312857580483 | [M-H]- | terpenoids |
| Caffeic Acid | C9H8O4 | 179.0350804 | 55.4554 | 0.44924486201167 | [M-H]- | phenylpro-panoids |
| Oleic acid | C18H34O2 | 281.2485305 | 752.812 | 1.6694653559302 | [M-H]- | Fatty acids |
| Licoricesaponin H2 | C42H62O16 | 821.3972517 | 387.5245 | 0.306444111480263 | [M-H]- | terpenoids |
| MANNITOL | C6H14O6 | 181.0720833 | 884.508 | 0.459994160865411 | [M-H]- | |
| MALEIC ACID | C4H4O4 | 115.0038273 | 37.67985 | 1.50173058643563 | [M-H]- | Organic acids and derivatives |
| 2,3-bis[(4-hydroxy-3-methoxyphenyl)methyl]butane-1,4-diol | C20H26O6 | 361.1658535 | 180.414 | 0.405639070227906 | [M-H]- | phenylpro-panoids |
| 3-phenyllactic acid | C9H10O3 | 165.0559929 | 107.996 | 0.0431100373257922 | [M-H]- | |
| (EZ)-sinapic acid | C11H12O5 | 223.0609987 | 105.679 | 0.00600471632465977 | [M-H]- | phenylpro-panoids |
| P-Anisic acid | C8H8O3 | 151.0406067 | 84.9642 | 4.01668965934979 | [M-H]- | Phenols |
| Kaempferide | C16H12O6 | 299.0560035 | 299.623 | 0.0118599257969557 | [M-H]- | flavonoids |
| Oleanolic acid base + O-HexA-Hex | C42H66O14 | 793.4382418 | 416.953 | 0.304810732901507 | [M-H]- | terpenoids |
| 2-METHYLMALEATE | C5H6O4 | 129.0196012 | 36.2266 | 3.09112915682575 | [M-H]- | Aliphatic acyl |
| 14-hydroxy-14-(hydroxymethyl)-5,9-dimethyltetracy-clo[11.2.1.0¹,¹⁰.0⁴,⁹]hexadecane-5-carboxylic acid | C20H32O4 | 335.2225099 | 472.74 | 1.46201775106086 | [M-H]- | terpenoids |
| Isofraxidin | C11H10O5 | 221.0455865 | 89.7378 | 1.87080486699862 | [M-H] | phenylpro-panoids |
| L-ISOLEUCINE | C6H13NO2 | 130.0875223 | 58.3162 | 3.67220756128329 | [M-H]- | Amino acid deriv-atives |
| 3,4,5-trimethoxycinnamic acid | C12H14O5 | 237.0769219 | 213.003 | 0.329265714068399 | [M-H]- | phenylpro-panoids |

| | | | | | | |
|---|---|---|---|---|---|---|
| Vanillin | C8H8O3 | 151.0403144 | 95.8936 | 2.08167347397003 | [M-H]- | Phenols |
| Sebacic acid | C10H18O4 | 201.1131245 | 214.073 | 0.618874607685535 | [M-H]- | Aliphatic acyl |
| 4-Hydroxyphenylacetic acid | C8H8O3 | 151.0404936 | 61.1463 | 3.26767616690824 | [M-H]- | Phenols |
| Abscisic acid | C15H20O4 | 263.1287834 | 313.846 | 0.823038723344125 | [M-H]- | terpenoids |
| Hexadecanedioic acid | C16H30O4 | 285.2076122 | 344.027 | 2.1466308392147 | [M-H]- | |
| Austricine | C15H18O4 | 261.1134886 | 273.61 | 1.87125003617752 | [M-H]- | alkaloid |
| 9-Hydroxy-10,12,15-octadecatrienoic acid | C18H30O3 | 293.2128447 | 605.97 | 2.88097922089447 | [M-H]- | |
| Piperonylic Acid | C8H6O4 | 165.0196619 | 59.3518 | 2.04909254577965 | [M-H]- | Organic acids and derivatives |
| (R,R)-TARTARIC ACID | C4H6O6 | 149.0092255 | 43.3027 | 1.51309554356264 | [M-H]- | |
| Piscidic Acid | C11H12O7 | 255.0509923 | 38.3427 | 0.0302708133833345 | [M-H]- | |
| Saikosaponin a | C42H68O13 | 825.4641441 | 422.351 | 2.24835689632638 | [M+HCOO]- | terpenoids |
| Gallic acid | C7H6O5 | 169.0144523 | 68.7533 | 3.24038018901549 | [M-H]- | Phenols |
| Piceatannol | C14H12O4 | 243.066471 | 143.557 | 1.93772370195578 | [M-H] | Phenols |
| adipic acid | C6H10O4 | 145.0507244 | 43.97955 | 1.89994565083979 | [M-H]- | Fatty acids |
| Bayogenin base + O-Hex-Hex | C42H68O15 | 857.4515226 | 278.277 | 4.05552535792022 | [M+HCOO]- | terpenoids |
| Azelaic acid | C9H16O4 | 187.0973113 | 155.648 | 1.66395626849927 | [M-H]- | Aliphatic acyl |
| 5,9-dihydroxy-5,7,7-trimethyl-4,5a,6,8,8a,9-hexahydro-1H-azuleno[5,6-c]furan-3-one | C15H22O4 | 265.1440808 | 376.054 | 0.304716860852492 | [M-H]- | terpenoids |
| 4-Methylcatechol | C7H8O2 | 123.0453102 | 40.8227 | 2.52067018730777 | [M-H]- | Phenols |
| Medicagenic acid | C30H46O6 | 501.3218812 | 421.759 | 2.23170111242219 | [M-H]- | terpenoids |
| Atractylenolide III | C15H20O3 | 247.1335113 | 394.07 | 1.97758691027067 | [M-H]- | terpenoids |
| 3,12-dihydroxy-4,6a,6b,11,12,14b-hexamethyl-1,2,3,4a,5,6,7,8,9,10,11,12a,14,14a-tetradecahydropicene-4,8a-dicarboxylic acid | C30H46O6 | 501.3216077 | 434.695 | 2.7771836032294 | [M-H]- | terpenoids |
| Oleanane -2H, +1O, 1COOH, O-HexA-HexA | C42H64O15 | 807.4179258 | 378.421 | 0.0918533941399496 | [M-H]- | terpenoids |
| Coumaroyl quinic acid | C16H18O8 | 337.0928305 | 55.4554 | 0.502709721598067 | [M-H]- | phenylpro-panoids |
| Suberic acid | C8H14O4 | 173.0819491 | 97.6209 | 0.294275903879789 | [M-H]- | Fatty acids |
| Phenylethyl primeveroside | C19H28O10 | 461.1663575 | 83.47235 | 1.39310269593394 | [M+FA-H]- | alkaloid |
| Flavone base + 3O, C-Hex-dHex | C27H30O14 | 577.1583698 | 89.7378 | 2.37326543415683 | [M-H]- | flavonoids |
| SUCROSE | C12H22O11 | 341.1093574 | 578.388 | 1.04765792056787 | [M-H]- | Carbohy-drates and derivatives |
| [6-[3,4-dihydroxy-2,5-bis(hydroxymethyl)oxolan-2-yl]oxy-3,4,5-trihydroxyoxan-2-yl]methyl (E)-3-(4-hydroxy-3-methoxyphenyl)prop- | C22H30O14 | 517.1566702 | 46.63115 | 0.637652485299422 | [M-H]- | phenylpro-panoids |

| | | | | | | |
|---|---|---|---|---|---|---|
| 2-enoate | | | | | | |
| 4-[2-(2,6-dimethoxy-4-prop-2-enylphenoxy)-1-hydroxypropyl]-2-methoxyphenol | C21H26O6 | 373.1682589 | 311.529 | 1.98600604244762 | [M-H]- | |
| 4-[4-[hydroxy-(4-hydroxy-3-methoxyphenyl)methyl]-3-(hydroxymethyl)oxolan-2-yl]-2-methoxyphenol | C20H24O7 | 375.144928 | 242.789 | 0.192006322431133 | [M-H]- | phenylpro-panoids |
| Benzyl alcohol + Hex-Pen | C18H26O10 | 447.1506618 | 126.449 | 0.756402599716079 | [M+HCOO]- | |
| 5-[(Z)-5-hydroxy-3-methylpent-3-enyl]-1,4a-dimethyl-6-methylidene-3,4,5,7,8,8a-hexahydro-2H-naphthalene-1-carboxylic acid | C20H32O3 | 319.2282712 | 482.208 | 0.849481112622956 | [M-H]- | terpenoids |
| 1,3-Dicaffeoylquinic acid | C25H24O12 | 515.1235216 | 63.4597 | 0.928708140847708 | [M-H] | phenylpro-panoids |
| Hispidulin | C16H12O6 | 299.0558747 | 288.295 | 0.418887902057755 | [M-H] | flavonoids |
| Zizyberanalic acid | C42H64O16 | 823.4151107 | 397.952 | 2.56340159472414 | [M-H]- | terpenoids |
| D-Gluconic acid | C6H12O7 | 195.0510508 | 32.5537 | 0.260575960962236 | [M-H]- | Organic acids and derivatives |
| isoferulic acid | C10H10O4 | 193.0507754 | 107.412 | 1.16350186394557 | [M-H]- | phenylpro-panoids |
| Pectolinarin | C29H34O15 | 621.1820556 | 206.059 | 1.52033943749386 | [M-H]- | flavonoids |
| Inositol | C6H12O6 | 179.0562558 | 36.1272 | 1.42849263133504 | [M-H]- | |
| Grandisin | C24H32O7 | 431.2094906 | 588.957 | 1.18126029232946 | [M-H]- | phenylpro-panoids |
| Glutathione | C10H17N3O6S | 306.0771248 | 40.1392 | 0.407758793138918 | [M-H]- | alkaloid |
| Flavonol base + 3O, O-Hex-Hex | C27H30O16 | 609.1461847 | 87.9407 | 2.98008653039655 | [M-H]- | flavonoids |
| Formononetin | C16H12O4 | 267.066041 | 310.415 | 0.153657162306396 | [M-H] | flavonoids |
| Dihydrocapsaicin | C18H29NO3 | 306.2077278 | 532.104 | 0.88885872000212 | [M-H]- | alkaloid |
| 5,7-dihydroxy-2-(4-hydroxyphenyl)-6-[3,4,5-trihydroxy-6-(hydroxymethyl)oxan-2-yl]-4H-chromen-4-one | C21H20O10 | 431.0987625 | 95.2172 | 0.550882423294907 | [M-H]- | flavonoids |
| Pogostone | C12H16O4 | 223.0979607 | 167.496 | 0.176250710139899 | [M-H] | |

**Supplementary Table 5. Positive ion mode composition table**

| Name | Formula | mzmed | rtmed | ppm | ms2Adduct | Class |
|---|---|---|---|---|---|---|
| (R)-ar-Turmerone | C15H20O | 217.158703 | 469.862 | 1.36770300664146 | [M+H]+ | |
| 3-Methoxybenzaldehyde | C8H8O2 | 137.0597395 | 60.309 | 1.90087166383438 | [M+H]+ | |
| 7-methyl-3-methylidene-6-(3-oxobutyl)-4,7,8,8a-tetrahydro-3aH-cyclohepta[b]furan-2-one | C15H20O3 | 271.1306156 | 336.169 | 2.27060401718192 | [M+Na]+ | |
| 7,8-Dihydroxycoumarin | C9H6O4 | 179.0337515 | 952.084 | 1.388243345043 | [M+H] | phenylpro nylpro-panoids |
| 9-Hydroxycalabaxanthone | C24H24O6 | 409.1621995 | 399.712 | 0.48768971880123 | [M+H]+ | |

| | | | | | | |
|---|---|---|---|---|---|---|
| Acetophenone | C8H8O | 121.064461 | 683.682 | 4.45233128395336 | [M+H] | Phenols |
| Adenine | C5H5N5 | 136.0616317 | 32.4079 | 2.70709339952972 | [M+H] | alkaloid |
| Chaulmoogric Acid | C18H32O2 | 298.2735665 | 605.032 | 1.45341839737744 | [M+NH4]+ | Fatty acids |
| Curcumenol | C15H22O2 | 257.1503677 | 896.871 | 1.4298576250763 | [M+Na]+ | terpenoids |
| Fraxetin | C10H8O5 | 209.0441151 | 11.677835 | 0.550785674640299 | [M+H] | phenylpro nylpro-panoids |
| Glyceryl linolenate | C21H36O4 | 375.250309 | 652.505 | 0.823563255078567 | [M+Na]+ | |
| Isopeonol | C9H10O3 | 167.070079 | 98.2412 | 0.473080676381678 | [M+H]+ | Phenols |
| Loureirin A | C17H18O4 | 287.1250134 | 351.278 | 0.0466235138117437 | [M+H] | flavo-noids |
| Lucidone C | C24H36O5 | 405.2599562 | 609.244 | 2.57568928811573 | [M+H]+ | terpenoids |
| Nicotinic acid | C6H5NO2 | 124.039278 | 34.0151 | 2.24126186055438 | [M+H] | alkaloid |
| p-Hydroxybenzaldehyde | C7H6O2 | 123.0439339 | 50.2229 | 0.5374865539597 | [M+H] | Phenols |
| Perilloside A | C16H26O6 | 315.1795935 | 667.62 | 1.28961146061034 | [M+H]+ | |
| Tyramine | C8H11NO | 121.0644227 | 606.33 | 4.76819114093064 | [M+H-NH3]+ | alkaloid |
| Valine | C5H11NO2 | 118.086124 | 792.363 | 1.05020330742182 | [M+H]+ | alkaloid |
| 7-hydroxy-1,4a-dimethyl-9-oxo-7-propan-2-yl-2,3,4,4b,5,6,10,10a-octahydrophenanthrene-1-carboxylic acid | C20H30O4 | 335.2193422 | 473.185 | 1.02093707870274 | [M+H]+ | terpenoids |
| 1-Methyl-2-pyrrolecarboxaldehyde | C6H7NO | 110.0600072 | 32.4079 | 0.0652717020947082 | [M+H]+ | alkaloid |
| Betaine | C5H11NO2 | 118.0860356 | 884.757 | 0.301811580060181 | [M+H]+ | alkaloid |
| Coumaric acid | C9H8O3 | 147.0440164 | 50.3271 | 0.111381675927173 | [M+H-H2O]+ | phenylpro nylpro-panoids |
| 2-Acetylfuran | C6H6O2 | 111.043895 | 34.9222 | 0.945450634474365 | [M+H]+ | |
| Kaempferide | C16H12O6 | 301.0696544 | 382.011 | 1.14795907175651 | [M+H]+ | flavo-noids |
| Benzoic acid | C7H6O2 | 123.0439971 | 105.244 | 0.0238005516055592 | [M+H] | Phenols |
| Myristicin | C11H12O3 | 193.0857048 | 429.71 | 1.52899432586117 | [M+H]+ | |
| Indole | C8H7N | 118.0648619 | 42.9545 | 1.16998779159512 | [M+H]+ | alkaloid |
| 2-Hydroxyacetophenone | C8H8O2 | 137.0592479 | 509.59 | 1.80853139671337 | [M+H]+ | Phenols |
| Sesamol | C7H6O3 | 139.0390384 | 44.0955 | 0.275856561390893 | [M+H]+ | |
| Salicylic acid | C7H6O3 | 139.0387351 | 184.343 | 1.90536167370063 | [M+H]+ | Phenols |
| Tectochrysin | C16H12O4 | 269.0803636 | 311.04 | 2.36494595305206 | [M+H] | flavo-noids |
| 2,3-Secoporrigenin | C27H40O6 | 461.2863614 | 705.175 | 1.38442406591123 | [M+H]+ | terpenoids |
| 2-(3,7-Dimethyl-2,6-octadienyl)-4-hydroxy-6-methoxyacetophenone | C19H26O3 | 303.1956293 | 427.835 | 2.07569568659513 | [M+H]+ | |
| Flavanone base + 4O, 2Prenyl | C25H28O6 | 425.1927363 | 514.783 | 0.620270598401287 | [M+H]+ | flavo-noids |
| Acetylvanillin | C10H10O4 | 195.0650862 | 118.552 | 0.441677399541844 | [M+H] | Phenols |
| 2-Methylbenzaldehyde | C8H8O | 121.0644045 | 652.505 | 4.91900856027561 | [M+H]+ | |

| | | | | | | |
|---|---|---|---|---|---|---|
| Methyl 2-furoate | C6H6O3 | 127.0388784 | 488.8075 | 0.957158096192188 | [M+H]+ | |
| 3,4-Dimethylbenzoic acid | C9H10O2 | 151.075082 | 263.019 | 0.542922531713652 | [M+H]+ | |
| p-Mentha-1,3,8-triene | C10H14 | 135.1167518 | 230.48 | 1.83683885715142 | [M+H]+ | |
| Batatasin IV | C15H16O3 | 245.1166858 | 265.217 | 1.28177586498756 | [M+H]+ | |
| Esculetin | C9H6O4 | 179.0331088 | 1.67307 | 0.607545100167653 | [M+H] | phenylpro nylpro-panoids |
| Glutamate | C5H9NO4 | 148.0602934 | 32.4079 | 1.98141853785394 | [M+H]+ | Amino acid de-rivatives |
| Methyl phenylacetate | C9H10O2 | 151.0751413 | 192.4 | 0.935210795211156 | [M+H]+ | |
| p-Mentha-1,3,5,8-tetraene | C10H12 | 133.1010477 | 168.231 | 0.358291867976389 | [M+H]+ | |
| 4-Coumaryl alcohol | C9H10O2 | 151.0755191 | 83.5044 | 3.43585126954599 | [M+H]+ | |
| 3,4,5-trimethoxycinnamic acid | C12H14O5 | 239.0914775 | 213.489 | 1.99725454852997 | [M+H]+ | phenylpro nylpro-panoids |
| Lucidenic acid M | C27H42O6 | 463.3029401 | 656.213 | 2.02907290812967 | [M+H]+ | terpenoids |
| 4-[2-(2,6-dimethoxy-4-prop-2-enylphenoxy)-1-hydroxypropyl]-2-methoxyphenol | C21H26O6 | 397.1622411 | 353.924 | 0.607034040694588 | [M+Na]+ | |
| 3',4',7-Trihydroxyflavanone | C15H12O5 | 273.0755235 | 278.015 | 1.74487675025091 | [M+H]+ | flavo-noids |
| 4-Methoxysalicylic acid | C8H8O4 | 169.0489762 | 1.91917 | 0.140775546446282 | [M+H] | Phenols |
| (-)-3-Thujene | C10H16 | 137.1319035 | 24.53785 | 0.703891505599481 | [M+H]+ | terpenoids |
| Azuleno(5,6-c)furan-1(3H)-one, 4,4a,5,6,7,7a,8,9-octahydro-3,4,8-trihydroxy-6,6,8-trimethyl- | C15H22O5 | 283.1536362 | 156.234 | 1.28479456195822 | [M+H]+ | terpenoids |
| Maltol | C6H6O3 | 127.0387969 | 389.111 | 1.59858987907851 | [M+H]+ | flavo-noids |
| Coniferyl aldehyde | C10H10O3 | 179.0702392 | 357.208 | 1.33584941343721 | [M+H]+ | phenylpro nylpro-panoids |
| Abscisic acid | C15H20O4 | 265.1434626 | 263.6 | 1.74455839335035 | [M+H] | terpenoids |
| Coumaroyl tyramine | C17H17NO3 | 284.1283791 | 178.733 | 1.33415064442162 | [M+H]+ | |
| PHENYLALANINE | C9H11NO2 | 166.0864808 | 82.3192 | 2.89474203885818 | [M+H]+ | Amino acid de-rivatives |
| Benzofuran | C8H6O | 119.0488754 | 150.464 | 1.0464163526142 | [M+H]+ | |
| Isoleucine | C6H13NO2 | 132.1018619 | 40.70815 | 1.04521448073659 | [M+H]+ | Amino acid de-rivatives |
| Parthenolide | C15H20O3 | 249.148023 | 180.978 | 0.0922864276650613 | [M+H] | terpenoids |
| Hordenine | C10H15NO | 166.1219615 | 1.91917 | 0.231961961380639 | [M+H]+ | alkaloid |
| Thermophillin | C8H8O4 | 169.048964 | 20.0473 | 0.212873212224352 | [M+H]+ | quinones |
| 3',4'-Dihydroxyacetophenone | C8H8O3 | 153.0544756 | 179.889 | 3.10766925308712 | [M+H]+ | |
| Pyroglutamic acid (not validated, isomer of 88) | C5H7NO3 | 130.0497407 | 33.227 | 1.99361849968787 | [M+H]+ | Amino acid de- |

| | | | | | | |
|---|---|---|---|---|---|---|
| | | | | | | rivatives |
| Anisic aldehyde | C8H8O2 | 137.0595191 | 43.635 | 3.787195384224 | [M+H] | Phenols |
| L-2,3-DIAMINOPROPIONIC ACID | C3H8N2O2 | 105.0696187 | 379.642 | 3.62923396684895 | [M+H]+ | |
| Cyclo(leucylprolyl) | C11H18N2O2 | 211.1438575 | 98.821 | 0.674864770838979 | [M+H]+ | |
| Ethyl vanillin | C9H10O3 | 167.0700914 | 228.71 | 0.547365187966084 | [M+H]+ | |
| Naphthalene-1,2-diol | C10H8O2 | 161.0593578 | 106.968 | 2.22127257923032 | [M+H]+ | |
| Licoricesaponin H2 | C42H62O16 | 823.4118011 | 352.352 | 0.972902134693928 | [M+H]+ | terpenoids |
| 6,7,8-trimethoxychromen-2-one | C12H12O5 | 237.0755884 | 171.4415 | 1.73595745009731 | [M+H]+ | phenylpro nylpro-panoids |
| Allyl benzoate | C10H10O2 | 163.0751031 | 577.859 | 0.632302540612125 | [M+H]+ | |
| Licochalcone C | C21H22O4 | 339.1593349 | 443.034 | 0.987417580963433 | [M+H] | flavo-noids |
| (S)-Curzeone | C15H16O2 | 229.1219115 | 466.673 | 0.386085082010296 | [M+H]+ | |
| kojic acid | C6H6O4 | 143.0338814 | 36.1538 | 0.829216664151295 | [M+H]+ | |
| 2'-Hydroxy-5'-methylacetophenone | C9H10O2 | 151.074984 | 320.673 | 0.105580285774118 | [M+H]+ | |
| Germacrone | C15H22O | 219.1743544 | 589.065 | 1.61710127958718 | [M+H] | terpenoids |
| 8-Desoxygartanin | C23H24O5 | 381.1667636 | 480.544 | 0.620324652036303 | [M+H] | Xantho-nes |
| 2-Propylphenol | C9H12O | 137.0960592 | 348.889 | 0.431612712626667 | [M+H]+ | |
| cuminyl alcohol | C10H14O | 133.1010343 | 316.106 | 0.257939002302054 | [M+H-H2O]- | Prenol lipids |
| 3-Hydroxy-5-isopropylidene-3,8-dimethyl-2,3,3a,4,5,8a-hexahydro-6(1H)-azulenone | C15H22O2 | 235.1686586 | 333.198 | 1.45165565442944 | [M+H]+ | terpenoids |
| 3-Formylindole | C9H7NO | 146.0599223 | 136.834 | 0.531813489762294 | [M+H]+ | alkaloid |
| trans-Cinnamaldehyde | C9H8O | 133.0646672 | 399.707 | 2.50120496331197 | [M+H]+ | phenylpro nylpro-panoids |
| 2-(8-hydroxy-4a,8-dimethyl-1,2,3,4,5,6,7,8a-octahydronaphthalen-2-yl)prop-2-enoic acid | C15H24O3 | 235.1694609 | 169.383 | 1.9598735788797 | [M-H2O+H]+ | terpenoids |
| Azuleno[5,6-c]furan-1(3H)-one, 4,4a,5,6,7,7a,8,9-octahydro-4,8-dihydroxy-6,6,8-trimethyl- | C15H22O4 | 249.1480217 | 222.55 | 0.0869839096513677 | [M-H2O+H]+ | terpenoids |
| 3-(1,1-Dimethylallyl)herniarin | C15H16O3 | 245.1166588 | 246.8605 | 1.39217504731374 | [M+H]+ | phenylpro nylpro-panoids |
| Methyl 2-aminobenzoate | C8H9NO2 | 152.0707412 | 64.3313 | 1.70166658557277 | [M+H]+ | |
| 3,4-Dimethoxybenzaldehyde | C9H10O3 | 167.0700837 | 156.299 | 0.50127647711405 | [M+H]+ | Aromatic-ity |
| Phthalic anhydride | C8H4O3 | 149.0228159 | 608.107 | 1.2350867068364 | [M+H]+ | |
| 2-Hydroxy-4-methoxybenzaldehyde | C8H8O3 | 153.0545575 | 95.9351 | 2.89087454909906 | [M+H] | Phenols |
| Ethyl p-anisate | C10H12O3 | 181.0860625 | 63.1779 | 0.345140140159588 | [M+H]+ | |
| Furohyperforin | C35H52O5 | 553.3877052 | 867.699 | 2.33970572856314 | [M+H]+ | terpenoids |
| 1,4,5-Naphthalenetriol | C10H8O3 | 177.0547202 | 63.1779 | 1.58054458388635 | [M+H]+ | |

| | | | | | | |
|---|---|---|---|---|---|---|
| 3-Thujanone | C10H16O | 153.1268511 | 6.86873 | 0.972485393410415 | [M+H]+ | terpenoids |
| Adenosine | C10H13N5O4 | 268.1035081 | 32.4079 | 1.83466445266318 | [M+H]+ | alkaloid |
| 3beta,6beta-Dihydroxynortropane | C7H13NO2 | 144.1017922 | 32.4079 | 1.44202251640537 | [M+H]+ | alkaloid |
| 7-Methoxy-4-methylcoumarin | C11H10O3 | 191.0702652 | 72.6245 | 1.38774334036404 | [M+H] | phenylpro nylpro-panoids |
| (R)-Campholenic aldehyde | C10H16O | 153.1271012 | 155.663 | 0.66073340514954 | [M+H]+ | |
| Loliolide | C11H16O3 | 197.1169168 | 135.092 | 0.421877174871302 | [M+H]+ | terpenoids |
| Reichsteins substance S | C21H30O4 | 347.2194472 | 558.626 | 1.28790920742519 | [M+H]+ | terpenoids |
| Phenethylacetate | C10H12O2 | 165.0907398 | 209.332 | 1.57595595179957 | [M+H]+ | Aromatic-ity |
| 1-Piperidinecarboxaldehyde | C6H11NO | 114.0912173 | 49.7587 | 1.90423980242363 | [M+H]+ | alkaloid |
| 1beta-Hydroxyalantolactone | C15H20O3 | 249.1478881 | 345.328 | 0.448973157353429 | [M+H]+ | terpenoids |
| Geranial | C10H16O | 153.1268605 | 28.9739 | 0.911000007087903 | [M+H]+ | |
| 5,8-Dihydroxy-3,5,8-trimethyl-4a,5,6,7,7a,8,9,9a-octahydroazuleno[6,5-b]furan-2(4H)-one | C15H22O4 | 267.1586294 | 253.649 | 1.38700534486575 | [M+H]+ | terpenoids |
| Piperanine | C17H21NO3 | 288.1589626 | 97.6621 | 0.129794654490867 | [M+H]+ | alkaloid |
| (E)-1-Propenyl 2-propenyl disulfide | C6H10S2 | 147.0283697 | 27.7013 | 2.51444470725385 | [M+H]+ | |
| Vulgarin | C15H20O4 | 265.1435996 | 106.391 | 2.26144417175146 | [M+H]+ | terpenoids |
| (S)-Bilobanone | C15H20O2 | 233.1531286 | 350.661 | 0.551373189014369 | [M+H]+ | |
| 1-(4-Methoxyphenyl)-2-propanone | C10H12O2 | 165.0911804 | 88.8148 | 1.09292255654425 | [M+H]+ | |
| Deoxyvasicinone | C11H10N2O | 187.0865966 | 89.71495 | 2.15611476867002 | [M+H]+ | alkaloid |
| (4Z,7Z)-5,9,9-Trimethyl-11-oxabicyclo[8.2.1]trideca-1(13),4,7-triene-6,12-dione | C15H18O3 | 247.1322446 | 352.945 | 3.05677249567767 | [M+H]+ | |
| Periplocymarin | C30H46O8 | 535.3241295 | 669.207 | 2.11001292433457 | [M+H] | terpenoids |
| Methoxyeugenol | C11H14O3 | 195.101651 | 169.383 | 1.78863057868423 | [M+H]+ | |
| Methyl gallate | C8H8O5 | 185.0439291 | 15.23065 | 0.383355635451445 | [M+H] | Phenols |
| Isoeugenol | C10H12O2 | 165.090753 | 105.817 | 1.4958661187656 | [M+H]+ | |
| Cearoin | C14H12O4 | 245.08079 | 289.0645 | 0.856968443021479 | [M+H]+ | Phenols |
| Withaphysacarpin | C28H40O7 | 489.2851638 | 256.487 | 1.70912113826226 | [M+H]+ | terpenoids |
| 2-(3,4-dihydroxyphenyl)-5,7-dihydroxy-2,3-dihydrochromen-4-one | C15H12O6 | 289.0699718 | 388.406 | 0.0975076962717346 | [M+H]+ | flavo-noids |
| alpha-Linolenic acid | C18H30O2 | 279.2317469 | 550.397 | 2.67468230390173 | [M+H] | Aliphatic acyl |
| 3,8a-Dihydroxy-5-isopropylidene-3,8-dimethyl-2,3,3a,4,5,8a-hexahydro-6(1H)-azulenone | C15H22O3 | 233.1531892 | 178.211 | 0.811357780194178 | [M-H2O+H]+ | terpenoids |
| Haplophyllidine | C18H23NO4 | 318.1700365 | 113.392 | 0.114669374242577 | [M+H]+ | alkaloid |
| Panaquinquecol 1 | C18H28O3 | 293.210632 | 441.079 | 1.25495361979228 | [M+H]+ | |
| Acetylpanaxydol | C19H26O3 | 303.1949843 | 161.38 | 0.0517556615473683 | [M+H]+ | |
| Zedoarol | C15H18O3 | 247.1327112 | 335 | 1.16843204829786 | [M+H]+ | terpenoids |
| Polyporusterone B | C28H44O6 | 477.3169975 | 685.397 | 2.10029292749129 | [M+H]+ | terpenoids |

| | | | | | | |
|---|---|---|---|---|---|---|
| Furanofukinin | C16H24O2 | 249.1845857 | 550.397 | 1.66263372533937 | [M+H]+ | terpenoids |
| 1a,5,7a-Trimethyl-2,2a,6,6a,7a,8,9,9a-octahydrobisoxireno[4,5:8,9]cyclodeca[1,2-b]furan-4(1aH)-one | C15H20O4 | 265.1435698 | 225.3505 | 2.14900797501706 | [M+H]+ | terpenoids |
| Austricine | C15H18O4 | 263.1271689 | 242.857 | 0.641844746517921 | [M+H]+ | alkaloid |
| Edulan I | C13H20O | 193.1582305 | 347.699 | 1.19333071860404 | [M+H]+ | |
| Gingerenone A | C21H24O5 | 357.1695706 | 418.924 | 1.59761125241259 | [M+H]+ | |
| Dibutyl phthalate | C16H22O4 | 279.1589164 | 608.107 | 3.28282604302122 | [M+H]+ | Aromatic-ity |
| 7-Methoxycoumarin | C10H8O3 | 177.0547799 | 171.143 | 1.24318740311664 | [M+H] | phenylpro nylpro-panoids |
| Grandisin | C24H32O7 | 415.2104198 | 487.8315 | 1.39729519617945 | [M-H2O+H]+ | phenylpro nylpro-panoids |
| Methylisoeugenol | C11H14O2 | 179.1063696 | 585.4445 | 2.06381611526108 | [M+H]+ | |
| 8-Hydroxy-7-methoxy-2H-1-benzopyran-2-one | C10H8O4 | 193.0494293 | 100.037 | 2.22398971317528 | [M+H]+ | phenylpro nylpro-panoids |
| Isofraxidin | C11H10O5 | 223.0599794 | 46.1025 | 0.0923341787544959 | [M+H]+ | phenylpro nylpro-panoids |
| N-cis-Feruloyltyramine | C18H19NO4 | 314.1390492 | 193.561 | 0.156643868804106 | [M+H]+ | |
| 1,2-Dehydro-alpha-cyperone | C15H20O | 217.1587687 | 444.3 | 1.06489331891563 | [M+H]+ | |
| 2,3-dihydroxypropyl hexadecanoate | C19H38O4 | 313.2734225 | 754.301 | 1.34857142897031 | [M-H2O+H]+ | |
| 4-Methoxycinnamic acid | C10H10O3 | 179.0700269 | 234.687 | 0.150080845390516 | [M+H]+ | phenylpro nylpro-panoids |
| Paeonol | C9H10O3 | 167.0700523 | 204.975 | 0.312972799599941 | [M+H] | Phenols |
| Methylparaben | C8H8O3 | 153.0545317 | 60.851 | 3.05952039927576 | [M+H]+ | Phenols |
| (2Z,6E,10E)-12-hydroxy-6,10-dimethyl-2-(4-methylpent-3-enyl)dodeca-2,6,10-trienoic acid | C20H32O3 | 303.2319107 | 370.559 | 0.294377764560443 | [M-H2O+H]+ | |
| Pesticide3_Propoxur_C11H15NO3_Baygon | C11H15NO3 | 210.1123943 | 45.6415 | 1.87679709351608 | [M+H]+ | |
| alpha-Asarone | C12H16O3 | 209.1176175 | 82.9107 | 2.95308466241858 | [M+H] | phenylpro nylpro-panoids |
| 5-Hydroxymethylfurfural | C6H6O3 | 127.0392275 | 74.4123 | 1.79057130249398 | [M+H] | |
| 2-Hexyl-5-[2-(4-hydroxy-3-methoxyphenyl)ethyl]furan | C19H26O3 | 303.1928243 | 553.844 | 0.579604891453249 | [M+H]+ | |
| Scoparone | C11H10O4 | 207.0651178 | 166.463 | 0.568699756309343 | [M+H] | phenylpro nylpro-panoids |
| Piceatannol | C14H12O4 | 245.0809438 | 133.961 | 0.229411814354426 | [M+H] | Phenols |
| 2,8-Dihydroxy-5,5,8-trimethyl-11-oxatetracyclo[7.3.1.0~1,9~.0~3,7~]tridecan-10-one | C15H22O4 | 267.1586099 | 116.838 | 1.46031533550186 | [M+H]+ | terpenoids |

| | | | | | | |
|---|---|---|---|---|---|---|
| Coumarin | C9H6O2 | 147.043958 | 305.5785 | 0.285408013765114 | [M+H] | phenylpro nylpro-panoids |
| alpha-Hydrojuglone 4-O-b-D-glucoside | C16H18O8 | 339.1070187 | 156.299 | 0.0550147355904896 | [M+H]+ | |
| Arginine | C6H14N4O2 | 175.1188837 | 36.2856 | 0.66408450949181 | [M+H]+ | Amino acid de-rivatives |
| Acorusdiol | C15H24O3 | 253.1791718 | 225.315 | 3.27121333131813 | [M+H]+ | terpenoids |
| Rishitin | C14H22O2 | 223.1691487 | 265.853 | 0.666460748914321 | [M+H]+ | |
| 1alpha-Hydroxyarbusculin A | C15H22O4 | 267.1585015 | 393.288 | 1.8659744392888 | [M+H]+ | terpenoids |
| Kaurane-17,18-dioic acid | C20H30O4 | 335.2193484 | 485.798 | 1.03919360179383 | [M+H]+ | terpenoids |
| Procurcumadiol | C15H22O3 | 251.1643847 | 174.673 | 1.53150218142049 | [M+H]+ | terpenoids |
| Dihydrovaltrate | C22H32O8 | 425.2136757 | 676.58 | 0.762753957672336 | [M+H]+ | |
| Di(2-ethylhexyl)phthalate (DEHP) | C24H38O4 | 391.2840432 | 807.895 | 0.110286922622159 | [M+H]+ | |
| Syringaldehyde | C9H10O4 | 183.0652927 | 107.8755 | 1.59888599142271 | [M+H]+ | |
| Bufalin | C24H34O4 | 387.2566819 | 568.765 | 1.76082130258859 | [M+H] | terpenoids |
| Ganolucidic acid A | C30H44O6 | 501.3215342 | 350.661 | 1.06568107192345 | [M+H]+ | terpenoids |
| Dehydrodiisoeugenol | C20H22O4 | 327.1585851 | 175.4765 | 1.26809308035073 | [M+H] | phenylpro nylpro-panoids |
| Irisflorentin | C20H18O8 | 387.1079032 | 355.343 | 2.33322846032685 | [M+H] | flavo-noids |
| Benzenepropanamide, N-[2-(acetyloxy)-1-(phenylmethyl)ethyl]-alpha-(benzoylamino)- | C27H28N2O4 | 445.2113246 | 444.3 | 1.5170476865264 | [M+H]+ | |
| Pterosin O | C15H20O2 | 233.1530604 | 497.099 | 0.259082698345419 | [M+H]+ | |
| Isoelemicin | C12H16O3 | 209.1169332 | 653.073 | 0.319302898913071 | [M+H]+ | |
| Polyporusterone C | C28H44O6 | 477.3199988 | 161.95 | 2.09763492127802 | [M+H]+ | terpenoids |
| Phenacetin | C10H13NO2 | 180.1018659 | 272.4815 | 0.744375669345183 | [M+H]+ | |
| Flavone base + 3O, C-Hex-dHex | C27H30O14 | 579.1696267 | 89.9939 | 2.37112714261019 | [M+H]+ | flavo-noids |
| 4-Nitrophenol | C6H5NO3 | 140.0340647 | 33.227 | 0.462020388730144 | [M+H]+ | Phenols |
| Gravacridonediol | C19H19NO5 | 342.1333902 | 133.3655 | 1.14042952030075 | [M+H]+ | alkaloid |
| Curcolone | C15H18O3 | 247.1330857 | 54.5655 | 0.346597723909778 | [M+H]+ | |
| (1alpha,6alpha,7alphaH)-2,4(15)-Copadiene | C15H22 | 203.1791435 | 350.0345 | 0.706058237843897 | [M+H]+ | terpenoids |
| Zizyberanalic acid | C30H46O4 | 453.3369807 | 352.352 | 2.16336924553154 | [M-H2O+H]+ | terpenoids |
| Bisacurone epoxide | C15H24O4 | 269.1743516 | 291.705 | 2.40869344004561 | [M+H]+ | terpenoids |
| N-trans-Feruloyl-4-O-methyldopamine | C19H21NO5 | 344.1485129 | 325.408 | 1.4152748993558 | [M+H]+ | |
| 5-Hydroxyindoleacetic acid | C10H9NO3 | 192.065401 | 174.673 | 3.11896178608533 | [M+H]+ | alkaloid |
| 1-Hydroxyepiacorone | C15H24O3 | 253.1800875 | 126.347 | 0.345574297910582 | [M+H]+ | terpenoids |
| (3,6,9-trimethylidene-2-oxo-3a,4,5,6a,7,8,9a,9b-octahydroazuleno[4,5-b]furan-8-yl) acetate | C17H20O4 | 289.1425399 | 286.411 | 1.59119608315731 | [M+H]+ | terpenoids |
| Erysopine | C17H19NO3 | 286.1432028 | 361.435 | 2.78605723726414 | [M+H]+ | alkaloid |
| Gerberinol | C21H16O6 | 365.10626 | 746.661 | 3.45104992711081 | [M+H]+ | |

| | | | | | | |
|---|---|---|---|---|---|---|
| trans-Grandmarin | C15H16O6 | 293.1016954 | 263.019 | 1.03913680381112 | [M+H]+ | phenylpro nylpro-panoids |
| 3-Furfuryl 2-pyrrolecarboxylate | C10H9NO3 | 192.0653664 | 105.244 | 1.90793114229975 | [M+H] | alkaloid |
| Isoamberboin | C15H20O4 | 265.143523 | 488.481 | 1.97257808552382 | [M+H]+ | terpenoids |
| Phenylethyl primeveroside | C19H28O10 | 434.2018311 | 84.33465 | 0.388926630239522 | [M+NH4]+ | alkaloid |
| Isovanillic acid | C8H8O4 | 169.0495352 | 65.5051 | 2.74950388041365 | [M+H] | Phenols |
| Ganolucidic acid D | C30H44O6 | 501.3212111 | 298.301 | 0.421086395873976 | [M+H]+ | terpenoids |
| 4'-O-Methylglabridin | C21H22O4 | 339.1592019 | 353.924 | 0.595202839456823 | [M+H]+ | |
| 2-Cyclohexen-1-one, 4-hydroxy-4-(3-hydroxybutyl)-3,5,5-trimethyl- | C13H22O3 | 209.153291 | 768.272 | 1.39116346509882 | [M-H2O+H]+ | |
| Petroselinic acid | C18H34O2 | 283.2622938 | 636.59 | 2.49325333646355 | [M+H]+ | |
| 5-O-Methylembelin | C18H28O4 | 309.206 | 542.916 | 6.06262271787655e-05 | [M+H]+ | quinones |
| Dihydrocapsaicin | C18H29NO3 | 308.2222056 | 242.857 | 0.667211087515167 | [M+H] | alkaloid |
| 1,6-dihydroxy-5-methoxyxanthen-9-one | C14H10O5 | 259.0595234 | 344.131 | 1.83967041907062 | [M+H]+ | |
| Melilotocarpan C | C18H18O6 | 331.1176277 | 393.288 | 1.89573273496725 | [M+H]+ | |
| 1-Methoxy-3-carbaldehyde | C10H9NO2 | 176.0703649 | 41.64495 | 2.07238629415074 | [M+H]+ | alkaloid |
| 3,4-Dihydrocoumarin | C9H8O2 | 149.0598027 | 86.4632 | 1.32394188423827 | [M+H]+ | terpenoids |
| 9-Hydroxy-3',4'-dimethoxy-3,4-methylenedioxy-9,9'-epoxylignan | C21H24O6 | 373.1640499 | 342.918 | 0.133661707285777 | [M+H]+ | phenylpro nylpro-panoids |
| 4,4,8,10,14-pentamethyl-17-(4,5,6-trihydroxy-6-methylheptan-2-yl)-2,5,6,7,9,15-hexahydro-1H-cyclopenta[a]phenanthrene-3,16-dione | C30H46O5 | 469.3297232 | 296.496 | 2.72043484106699 | [M-H2O+H]+ | terpenoids |
| blennin C | C15H22O3 | 251.1637294 | 226.995 | 1.07748161589175 | [M+H]+ | terpenoids |
| [6-[3,4-dihydroxy-2,5-bis(hydroxymethyl)oxolan-2-yl]oxy-3,4,5-trihydroxyoxan-2-yl]methyl (E)-3-(4-hydroxy-3-methoxyphenyl)prop-2-enoate | C22H30O14 | 536.1987566 | 50.7511 | 3.27598166255416 | [M+NH4]+ | phenylpro nylpro-panoids |
| Juzirine | C17H15NO3 | 282.1124231 | 98.821 | 1.4999172076864 | [M+H]+ | alkaloid |
| (R)-Pterosin B | C14H18O2 | 219.1377039 | 314.371 | 1.35106689857363 | [M+H]+ | |
| Gelsemine | C20H22N2O2 | 323.1717345 | 848.363 | 0.821630667896565 | [M+H] | alkaloid |
| Spathulenol | C15H24O | 221.1898714 | 345.328 | 0.581259241991049 | [M+H]+ | terpenoids |
| 6-O-Methylcodeine | C19H23NO3 | 314.1746521 | 401.534 | 1.10726717346632 | [M+H]+ | alkaloid |
| Curzerenone | C15H18O2 | 231.1379711 | 188.256 | 0.124856395751947 | [M+H]+ | |
| Cantharidin | C10H12O4 | 197.0809176 | 187.101 | 0.418048768964556 | [M+H]+ | terpenoids |
| Cadabicilone | C15H22O3 | 251.1634477 | 265.853 | 2.19900798087598 | [M+H]+ | terpenoids |
| Scutellarein | C15H10O6 | 287.0550208 | 277.498 | 0.072325023068092 | [M+H] | flavo-noids |
| Tangeritin | C20H20O7 | 395.1122091 | 266.427 | 0.529305681106993 | [M+Na]+ | flavo-noids |
| 6-Hydroxy-5a-methyl-3,9-bis(methylene)decahydronaphtho[1,2-b]furan-2(3H)-one | C15H20O3 | 231.1380126 | 336.169 | 0.0543027252594296 | [M-H2O+H]+ | terpenoids |

| | | | | | | |
|---|---|---|---|---|---|---|
| (R)-Juziphine | C18H21NO3 | 300.1588791 | 406.556 | 0.402651346925026 | [M+H]+ | alkaloid |
| Acoramone | C12H16O4 | 225.1121218 | 346.515 | 0.541041228788479 | [M+H]+ | |
| Isosativan | C17H18O4 | 287.1270544 | 393.288 | 0.189479041183176 | [M+H]+ | |
| Sativanone | C17H16O5 | 301.1069293 | 362.042 | 0.23480147131412 | [M+H]+ | |
| dihydrodamascenone | C13H20O | 193.1588578 | 230.48 | 0.73629532509937 | [M+H]+ | |
| Methionine | C5H11NO2S | 150.0546659 | 105.244 | 2.22644145028998 | [M+H]+ | alkaloid |
| Kanzonol K | C26H28O6 | 437.1938 | 464.12 | 1.8297729933136 | [M+H]+ | flavo-noids |
| Pogostone | C12H16O4 | 225.1120552 | 300.683 | 0.245017051478172 | [M+H] | |
| Murrayone | C15H14O4 | 259.0959007 | 211.751 | 0.383345976257383 | [M+H] | phenylpro nylpro-panoids |
| Spiro[furan-3(2H),1'(7'H)-naphthalene]-2,7'-dione, 5-(3-furanyl)-2',3',4,4',4'a,5,8',8'a-octahydro-4'a-hydroxy-2',5'-dimethyl- | C19H22O5 | 331.153822 | 147.531 | 0.537471830805747 | [M+H]+ | terpenoids |
| Auxin b | C18H30O4 | 311.2215203 | 292.304 | 1.54149707442586 | [M+H]+ | terpenoids |
| Asarylaldehyde | C10H12O4 | 197.0808533 | 204.728 | 0.744353469875455 | [M+H] | Phenols |
| Beta-Caryophyllene Alcohol | C15H26O | 240.2322354 | 745.5 | 0.979794608400766 | [M+NH4]+ | terpenoids |
| Isoliquiritigenin | C15H12O4 | 257.0809076 | 293.504 | 0.359352617280708 | [M+H] | flavo-noids |

**Supplementary Table 6. Behavioral scoring table of IDPN model rats.**

| Score | Stereotypical behavior | Movement Behavior |
|---|---|---|
| 0 | No stereotypical behavior | Quiet or normal activity |
| 1 | Rotational behavior | Over-excited |
| 2 | Excessive up-and-down movement of the head and neck | Increased exploratory behavior |
| 3 | Excessive up and down movements of the head and neck plus rotational behavior | Non-stop running |
| 4 | Head swinging to the side and excessive up and down movements of the neck | Non-stop running with startling jump |

**Supplementary Table 7. Weight gain of rats in each group（$\bar{x} \pm S$，n=10）.**

| Day | Blank | Model | Tiapride | CPYJT | *F* | *P* |
|---|---|---|---|---|---|---|
| Day 0(g) | 65.87±15.11 | 62.65±10.04 | 63.51±12.20 | 63.24±10.93 | 0.134 | 0.939 |
| Day 3(g) | 73.65±16.69 | 78.56±12.84 | 77.09±15.51 | 78.69±15.18 | 0.241 | 0.867 |
| Day 5(g) | 89.10±22.15 | 79.55±14.42 | 79.26±15.25 | 81.71±15.79 | 0.715 | 0.549 |
| Day 7(g) | 117.04±25.13 | $85.26\pm13.18^{\#\#}$ | $82.61\pm16.47^{\#\#}$ | $84.58\pm14.47^{\#\#}$ | 8.45 | ＜0.001 |
| Day 14(g) | 143.75±23.71 | $116.15\pm17.33^{\#\#}$ | $106.79\pm18.21^{\#\#}$ | $101.74\pm15.54^{\#\#}$ | 9.786 | ＜0.001 |
| Day 21(g) | 175.54±24.33 | 157.22±20.94 | $140.20\pm19.77^{\#\#}$ | $134.04\pm18.00^{\#\#*}$ | 7.969 | ＜0.001 |
| Day 28(g) | 223.46±28.58 | $186.15\pm26.32^{\#\#}$ | $169.16\pm22.29^{\#\#}$ | $162.81\pm24.45^{\#\#*}$ | 11.384 | ＜0.001 |
| Day 35(g) | 273.05±20.47 | $228.16\pm26.00^{\#\#}$ | $201.11\pm22.22^{\#\#*}$ | $192.68\pm23.57^{\#\#**}$ | 24.422 | ＜0.001 |

Note:Comparison with blank group $^{\#}$P<0.05, $^{\#\#}$P<0.01 Comparison with model group $^{*}$P<0.05, $^{**}$P<0.01(n=10 for each group).

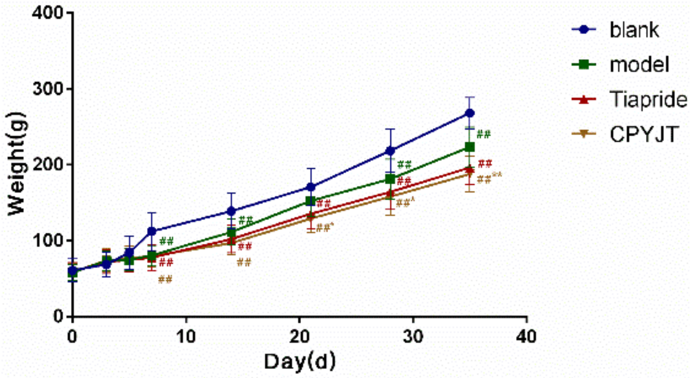


Supplementary Figure **1.** Weight gain of rats in each group($\bar{x} \pm S$, n=10)Comparison with blank group $^{\#}$P<0.05, $^{\#\#}$P<0.01. Comparison with model group $^{*}$P<0.05, $^{**}$P<0.01(n=10 for each group).

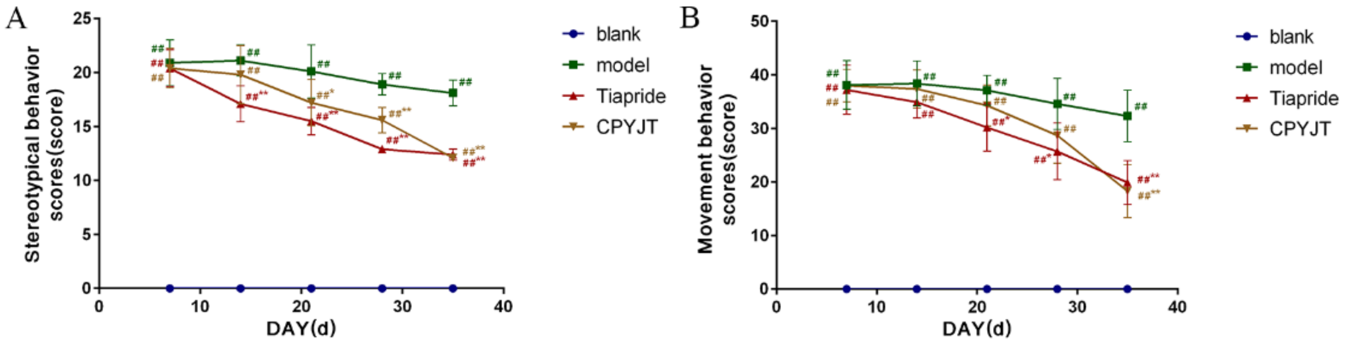


Supplementary Figure **2**. behavior scores of rats by each group ($\bar{x} \pm S$, n=10) (A) Stereotypical behavior; (B) Movement behavior; $^{\#\#}$P<0.01, $^{\#}$P<0.05 compared with the standard group; $^{**}$P<0.01, $^{*}$P<0.05 compared with the model group (n=10 for each group).
